# Mutual Monomer Orientation To Bias the Supramolecular Polymerization of [6]Helicenes and the Resulting Circularly Polarized Light and Spin Filtering Properties

**DOI:** 10.1021/jacs.2c00556

**Published:** 2022-04-11

**Authors:** Rafael Rodríguez, Cristina Naranjo, Anil Kumar, Paola Matozzo, Tapan Kumar Das, Qirong Zhu, Nicolas Vanthuyne, Rafael Gómez, Ron Naaman, Luis Sánchez, Jeanne Crassous

**Affiliations:** † Univ Rennes, CNRS, ISCR (Institut des Sciences Chimiques de Rennes) − UMR 6226, F-35000 Rennes, France; ‡ Departamento de Química Orgánica, Facultad de Ciencias Químicas, Universidad Complutense de Madrid, 28040 Madrid, Spain; § Department of Chemical and Biological Physics, Weizmann Institute of Science, Rehovot 76100, Israel; ∥ Aix Marseille Université, Centrale Marseille, CNRS, iSm2, UMR 7313, Marseille 13397, France

## Abstract

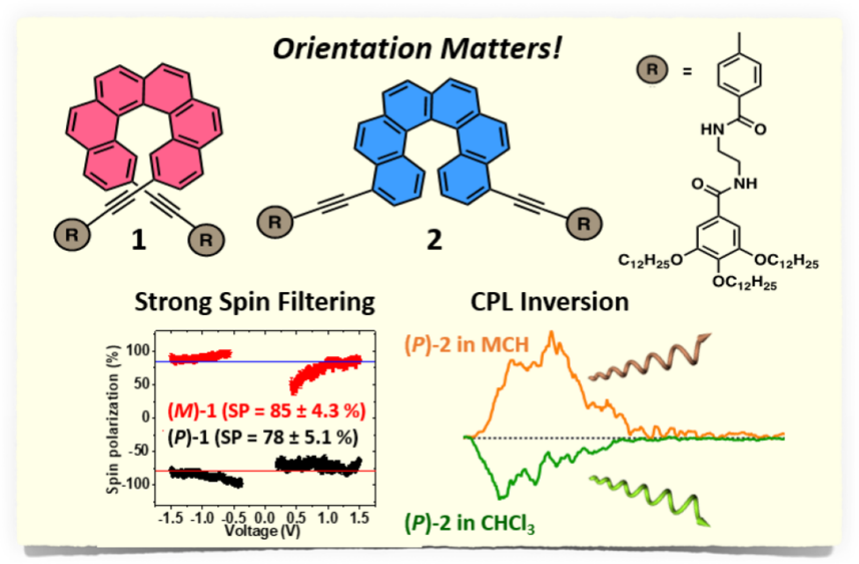

We report on the synthesis and self-assembly of 2,15- and 4,13-disubstituted carbo[6]helicenes **1** and **2** bearing 3,4,5-tridodecyloxybenzamide groups. The self-assembly of these [6]helicenes is strongly influenced by the substitution pattern in the helicene core that affects the mutual orientation of the monomeric units in the aggregated form. Thus, the 2,15-substituted derivative **1** undergoes an isodesmic supramolecular polymerization forming globular nanoparticles that maintain circularly polarized light (CPL) with *g*_lum_ values as high as 2 × 10^–2^. Unlike carbo[6]helicene **1**, the 4,13-substituted derivative **2** follows a cooperative mechanism generating helical one-dimensional fibers. As a result of this helical organization, [6]helicene **2** exhibits a unique modification in its ECD spectral pattern showing sign inversion at low energy, accompanied by a sign change of the CPL with *g*_lum_ values of 1.2 × 10^–3^, thus unveiling an example of CPL inversion upon supramolecular polymerization. These helical supramolecular structures with high chiroptical activity, when deposited on conductive surfaces, revealed highly efficient electron-spin filtering abilities, with electron spin polarizations up to 80% for **1** and 60% for **2**, as measured by magnetic conducting atomic force microscopy.

## Introduction

The development of new and enhanced technological applications in data storage, biological sensing, spintronic devices, and next-generation displays may benefit from the incorporation of efficient circularly polarized light (CPL) emitters^1−5^ and from excellent electron spin filtering due to the chirality-induced spin selectivity properties.^6^ Metal complexes,^2^ small organic molecules (pyrenes, binaphthyls, bodipys, etc.),^7^ and nanographene-based polycyclic aromatic hydrocarbons,^8^ decorated with stereogenic elements and efficient emissive features, are at the forefront of the research related to the development of new CPL emitters. In this regard, helicenes—*ortho*-fused aromatic compounds adopting helical chirality^9^—have recently emerged as appealing building blocks with efficient CPL activity^10^ and spin filtering capabilities^11^ that make them candidates for various spintronic applications. Beyond small molecules, the decoration or the coassembly of covalent polymers with CPL-emitting moieties has recently opened new avenues for the achievement of functional CPL emitters with a long range order.^12^ A crucial aspect for such achievement requires the organized arrangement of the chiral molecules into well-defined supramolecular aggregates. In this context, supramolecular polymers (SPs)^13^ constitute an excellent benchmark to investigate the formation of chiral supramolecular entities.^14^ Thus, the introduction of chirality in SPs usually results in the adoption of a macromolecular secondary helical structure.^14,15^ This property yielded new classes of innovative materials with stimuli-responsive nature.^16^ Since SPs are very often composed of achiral flat aromatic cores—such as BTAs,^17^ PBIs,^18^ π-conjugated oligomers,^19^ or porphyrins^20^—researchers have developed different strategies to achieve efficient transfer of asymmetry into the corresponding SP: (i) introducing point chirality in the side chains of the monomeric units,^17−20^ (ii) copolymerizing with nonracemic (Majority Rules, MR)^19,21^ or chiral/achiral monomeric mixtures (Sergeants and Soldiers effect, SaS),^17,21^ (iii) using chiral additives,^22^ or (iv) irradiating with CPL.^23^ Notably, a challenging strategy to obtain chiral SPs is based on the polymerization of nonplanar three-dimensional units and especially those involving axial or helical chirality that allows one to distinguish between homo- and heterochiral aggregation.^24,25^ Those chiral molecules present a highly distorted and rather rigid 3D-structure that precludes a straightforward self-assembly as denoted by the limited number of reports in the literature dealing with the controlled self-assembly of these systems in solution. In fact, very few examples of supramolecular polymers based on helicenes and stable in solution have been reported to date, among which is the formation of CPL-active helicene-based aggregates^26a,26b^ or the unique configurational stabilization of a [5]helicene system thanks to the formation of a chiral SP.^26c^ Very interestingly, chiral supramolecular polymers have recently proven to display highly efficient spin filtering^16b,27^ and were advantageously utilized for optimizing fundamental processes such as water splitting.^27a^ It is thus important to examine whether supramolecular polymers obtained from helicene building blocks display efficient spin selectivity and see how they compare with the reported self-assembled helicene systems.^11^ Furthermore, the combination of a helicene based on a supramolecular system that features both high CPL and high spin filtering properties was not demonstrated so far and is thus highly appealing in the context of spin-LED developments.

**Figure 1:**
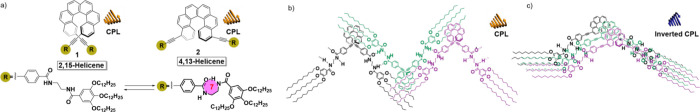
(a) Chemical structures of helicene derivatives **1** and **2**. Schematic illustration of the (b) head-to-tail self-assembly of **1** and the (c) head-to-head self-assembly of **2**. All the enantioenriched species, both in their monomeric or aggregated states, act as CPL emitters. The sign of the CPL emission of the aggregated species of helicene **2** is opposite to that registered for the monomeric species.

Herein, we report on the self-assembling features of configurationally stable 2,15- (compound **1**, Figure 1a) and 4,13-bis-ethynyl-carbo[6]helicene (compound **2**, Figure 1a) both in their racemic and enantiopure forms. Compounds **1** and **2** bear two peripheral N-(2-(4-ethynylbenzamido)ethyl)-3,4,5-tridodecyloxybenzamides to efficiently favor the supramolecular interaction of the reported [6]helicenes by the operation of a fourfold H-bonding array between the amides. We unveil dissimilar self-assembling and CPL emissive features for the enantioenriched forms of **1** and **2**. Thus, while [6]helicene **1** forms supramolecular aggregates in a head-to-tail fashion with no efficient overlap of the helicene backbones (Figure 1b), [6]helicene **2**, displaying a more favorable situation for the π-stacking interaction between the contorted helicene cores, forms head-to-head helical, supramolecular polymers in a cooperative manner (Figure 1c). Bis-ethynyl-[6]helicenes **1** and **2** exhibit CPL activity generated at the molecular level and with luminescence dissymmetry factors depending on the substitution pattern. Notably, the supramolecular polymerization of **2** allows one to bias the CPL sign; it becomes opposite in the aggregated state compared to the monomeric one (Figure 1c).^28^ In addition, we demonstrate very efficient spin filtering for the electrons transmitted through the supramolecular layer. Hence, these results represent an example of structure–function control in supramolecular polymers and pave the way to the development of stimuli-responsive CPL and spintronic materials.

## Results and Discussion

### Synthesis and Self-Assembly in Solution: Biasing the Supramolecular Polymerization Mechanism by the Substitution Pattern

The target chiral molecules **1** and **2** (Figure 1a) were readily prepared by following a double cross-coupling Sonogashira reaction between the racemic 2,15- and 4,13-bis-ethynyl-carbo[6]helicene building blocks^29^ and the iodo-bis(benzamide) derivative (see the Supporting Information (SI)).^30^ The enantioenriched (*P*) and (*M*) enantiomers of **1** and **2** were isolated by the high-performance liquid chromatography enantiomeric resolution process (see the Supporting Information for details). Standard spectroscopic techniques (proton nuclear magnetic resonance (^1^H NMR), ^13^C NMR, and Fourier transform infrared (FTIR) spectroscopy and high-resolution mass spectrometry–electrospray ionization mass spectrometry) have been used to corroborate the chemical structure of the newly described helicenes **1** and **2** (see the Supporting Information).

**Figure 2:**
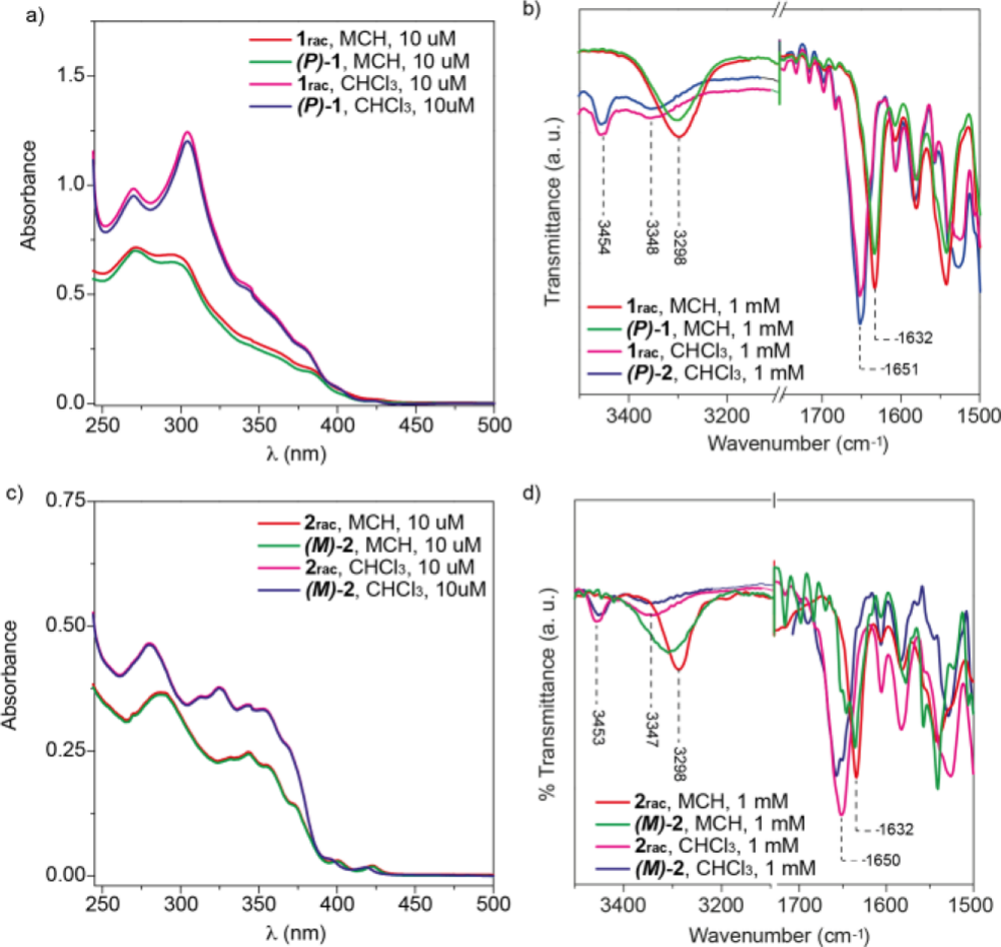
(a,c) UV–vis and (b,d) FTIR spectra of **1_rac_**, **(*P*)**-**1**, **2_rac_**, and **(*M*)-2** in CHCl_3_ and MCH (experimental conditions for UV–vis spectra: 298 K, *c*_T_ = 10 μM).

To unravel the self-assembly ability of [6]helicenes **1** and **2** and the formation of homochiral (conglomerates) or heterochiral (racemates) aggregates from the racemic mixture of the (*M*) and (*P*) enantiomers, we registered UV–vis spectra in CHCl_3_, a good solvent that favors the solvation of the monomeric species, and in methylcyclohexane (MCH), a bad solvent that usually provokes the efficient self-assembly of aromatic scaffolds. In the former solvent, both the racemic mixture of enantiomers of [6]helicene **1** (**1_rac_**) and the corresponding enantiomers **(*M*)-1** and **(*P*)-1**, at total concentration *c*_T_ = 10 μM, exhibit an identical absorption pattern with maxima at λ = 270 and 304 nm (Figure 2a). FTIR spectra in CHCl_3_ solution confirm that both **1_rac_** and **(*P*)-1** are in a molecularly dissolved state, since the stretching N–H and amide *I* bands appear at 3454 and 1651 cm^–1^, characteristic wavenumber values of free amides (Figure 2b).^31^ In addition, another stretching N–H band is observed at 3348 cm^–1^ that is ascribed to the formation of an intramolecular, 7-membered H-bonded pseudocycle (Figures 1a and 2b).^31,32^

The UV–vis spectra of **1_rac_** and **(*P*)-1** in MCH exhibit an identical absorption pattern, with maxima at λ = 270 and 299 nm (Figure 2a). These UV–vis spectra display a clear hypochromic effect but a very weak hypso- or bathochromic effect in comparison to those UV–vis spectra registered in CHCl_3_, thus suggesting a weak π-stacking of the contorted [6]helicene moiety (Figure 2a). In fact, concentration-dependent ^1^H NMR spectra recorded for both **1_rac_** and **(*M*)-1** in CDCl_3_ show no appreciable shift of the aromatic resonances but a clear deshielding of the resonances ascribable to the amide protons (Figure S3). However, the formation of intermolecular H-bonding arrays between the amide functional groups in MCH has been corroborated by using FTIR spectroscopy in this solvent. Thus, the stretching N–H and amide *I* bands appear at 3298 and 1632 cm^–1^, typical values of intermolecularly H-bonded amides (Figure 2b).^31^

The above-mentioned UV–vis spectra, in good agreement with that previously reported by Würthner and co-workers,^25^ suggest that **1_rac_** could be arranged as a conglomerate, constituted by an equal amount of homochiral, self-assembled (*M*) and (*P*) enantiomers. The formation of heterochiral aggregates (racemates) would afford different UV–vis spectra for both racemic **1_rac_** and the enantioenriched samples [**(*M*)-1** or **(*P*)-1**] due to the different electronic coupling between the chromophores.^25^

Identical findings have been obtained for [6]helicene **2**. Thus, the UV–vis spectra of the racemic mixture of enantiomers of **2** (**2_rac_**) and the (*M*) enantiomer **(*M*)-2** in CHCl_3_ show the same absorption pattern with maxima at λ = 280, 325, and 420 nm (Figure 2c). The stretching N–H and amide *I* bands, observed at 3453 and 1650 cm^–1^, confirm that **2_rac_** and **(*M*)-2** in CHCl_3_ are in the molecularly dissolved state (Figure 2d). In good analogy to compounds **1**, the stretching band observed at 3347 cm^–1^, together with the slight upfield shifts experienced by the amide protons upon heating a diluted solution of the helicenes in CDCl_3_ (Figure S7), is diagnostic of the formation of the metastable, intramolecularly H-bonded pseudocycle (Figures 2d and 1a).^31^ The UV–vis spectra of **2_rac_** and **(*M*)-2** in MCH present an identical absorption pattern diagnostic of the arrangement as conglomerates of the enantiomers in the racemic mixture of **2**.

The UV–vis spectra of **2_rac_** and **(*M*)-2** in MCH present a clear hypsochromic effect in comparison to those spectra recorded in CHCl_3_, and in addition, a weak but noticeable bathochromic shift is also detected (Figure 2c). This weak shift suggests the supramolecular interaction of the π-conjugated backbone of helicenes **2_rac_** and **(*M*)-2**. The π-stacking of the helicene cores and the operation of H-bonding interactions between the amide functional groups have also been corroborated by concentration-dependent ^1^H NMR experiments. These experiments show that the aromatic resonances for both **2_rac_** and **(*M*)-2** shift upfield, but the amide protons deshield upon increasing the concentration (Figure S8).

**Figure 3:**
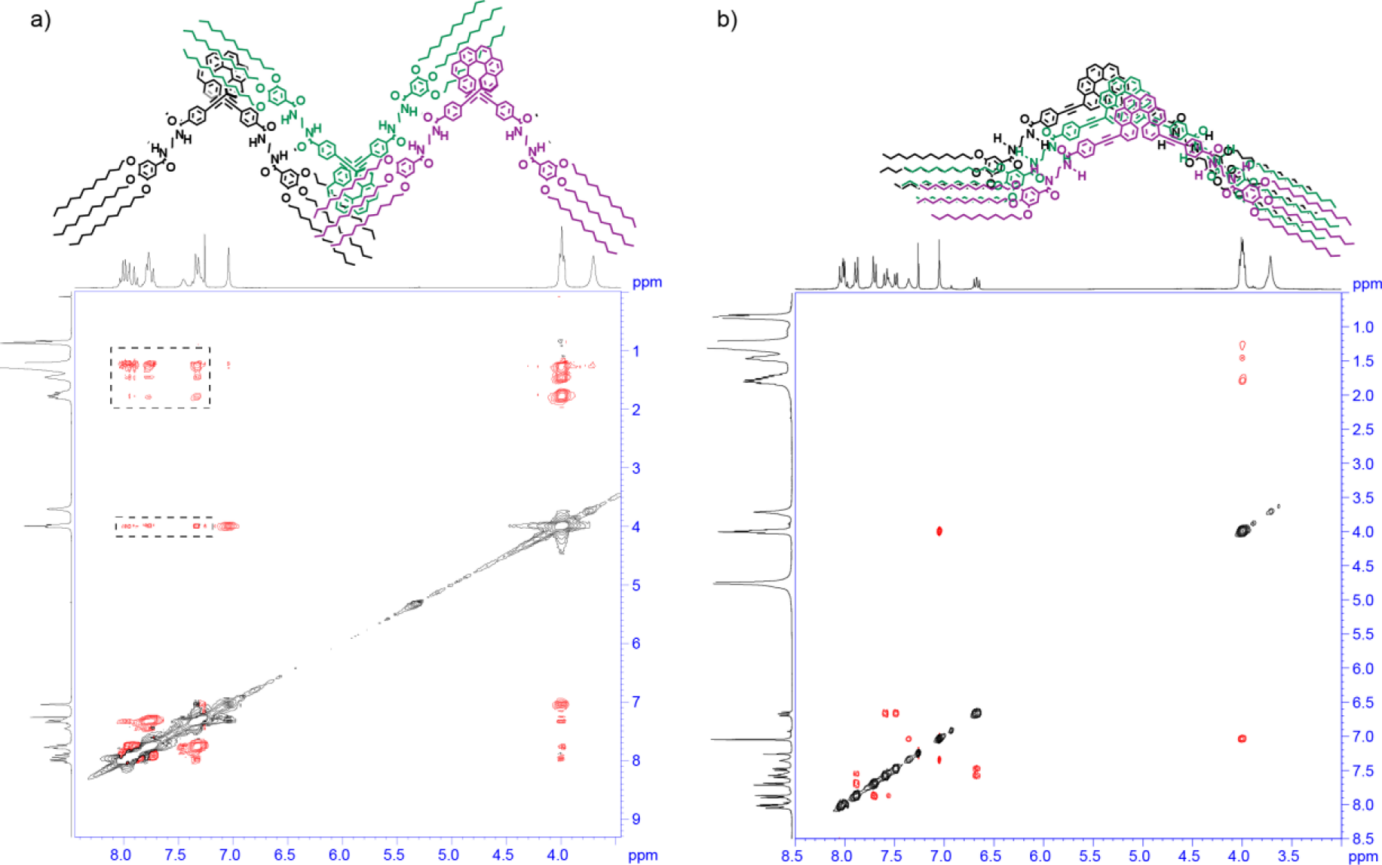
ROESY NMR spectra (CDCl_3_, 300 MHz, *c*_T_ = 20 mM; 293 K) of (a) **(*P*)-1** and (b) **(*M*)-2**. The dotted rectangles depict the intermolecular through-space coupling signals. The upper part of the panel depicts a schematic illustration of the binding mode experienced by the reported [6]helicenes upon self-assembly.

The spectroscopic studies carried out for **1** and **2** suggest very dissimilar self-assembling features of these two [6]helicenes due to the different substitution pattern. In the case of **1**, ROESY experiments, carried out in concentrated CDCl_3_ solutions of **(*P*)-1** (*c*_T_ = 20 mM), show the intermolecular contacts between the peripheral alkyl chains and most of the aromatic resonances that can only be justified by considering an alternate arrangement of the [6]helicene units interacting by the fourfold H-bonding array between the amide groups (Figures 1b and 3a).^33^

Unlike **1**, the ROESY experiments carried out on **(*M*)-2** (*c*_T_ = 20 mM) highlight the absence of any intermolecular interaction between the aromatic and aliphatic protons; this implies the stacking of the [6]helicene units, without an appreciable rotation angle between these stacked units, and with the four amides forming the fourfold H-bonding array (Figures 1c and 3b). Therefore, all the spectroscopic data (UV–vis, FTIR and ^1^H NMR) demonstrate that (i) although bulky, the [6]helicene can efficiently self-assemble, and (ii) the substitution pattern plays a crucial role in the self-assembling features. Indeed, while the 2,15-substitution pattern precludes the efficient interaction of the aromatic moieties but does not prevent the formation of intermolecular H-bonding interaction, the presence of the substituents at the 4 and 13 positions of the [6]helicene backbone favors both the π-stacking and the H-bonding between the amides. This substitution pattern plays a crucial role in both the supramolecular polymerization mechanism and has also a strong impact on the final chiroptical properties (vide infra).

### Supramolecular Polymerization Mechanism and Chiroptical and Emissive Properties

To unravel the supramolecular polymerization mechanism governing the self-assembly of [6]helicene **1**, we have initially performed variable-temperature (VT) UV–vis experiments by using MCH as the solvent. Heating up a diluted solution of **1_rac_** in MCH (*c*_T_ = 10 μM) results in an absorption pattern comparable to that observed in CHCl_3_ (Figures 2a and S4a). However, plotting the variation of the absorbance versus temperature yields an incomplete cooling curve that cannot be fitted to the one-component equilibrium model.^34^ Therefore, it is not possible to elucidate whether or not the supramolecular polymerization of this [6]helicene follows an isodesmic or a cooperative mechanism (Figure S4b).^13^ Identical findings have been achieved by registering VT-UV–vis spectra of enantioenriched **(*M*)-1** (Figure S4c and S4d).

**Figure 4:**
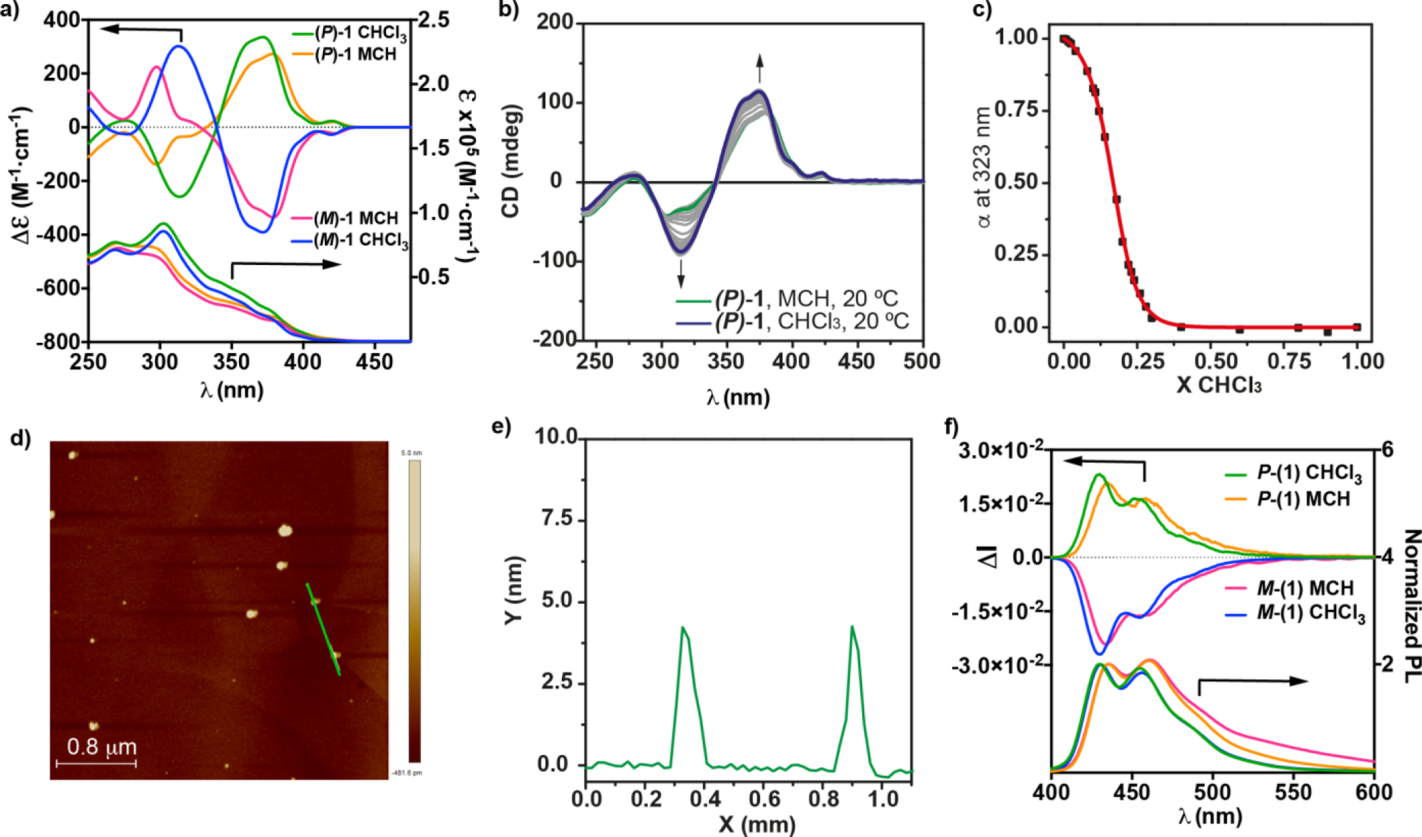
(a) ECD/UV–vis spectra of **(*P*)-1** and **(*M*)-1** in monomeric and aggregated states (CHCl_3_ and MCH, respectively). (b) CD spectra and (c) denaturation curve of **(*P*)-1** in MCH/CHCl_3_ mixtures. The red line in panel (c) depicts the fit to the SD model. (d) AFM image and (e) height profile of the globular supramolecular aggregates formed from **(*P*)-1** (experimental conditions: HOPG as the surface; *c*_T_ = 10 μM, and MCH as the solvent). (f) CPL/PL spectra of **(*P*)-1** and **(*M*)-1** in monomeric and aggregated states (CHCl_3_ and MCH, respectively) (experimental conditions for UV–vis, ECD, CPL, and PL spectra: *c*_T_ = 10 μM and λ_exc_ = 365 nm).

We have investigated the chiroptical properties of both enantiomers of [6]helicene **1** in CHCl_3_. **(*P*)-1** and **(*M*)-1** display mirror-image electronic circular dichroism (ECD) spectra and a pattern with maxima at 420, 375, 315, and 275 nm with zero crossing points at 340, 286, and 263 nm (Figure 4a). The findings obtained in the VT-UV–vis experiments and the changes observed in the ECD spectra, even if weak, are sufficient to utilize the solvent denaturation (SD) protocol, described by Meijer and co-workers,^35^ allowing one to derive a complete set of thermodynamic parameters associated with the supramolecular polymerization of **(*P*)-1**. This model recognizes the supramolecular polymerization as a balance between the effect of mixing a good and a bad solvent that favors the solvation or the aggregation of the monomeric species, respectively. To perform this study, solutions of ***(P)-*1** in MCH, as the bad solvent, and in CHCl_3_, as the good solvent, are mixed together keeping constant *c*_T_ = 10 μM. In this model, the Gibbs free energy increases upon monomer addition in a mixture of solvents (Δ*G*^0^′), and the Gibbs free energy in a pure solvent (Δ*G*^0^) is linearly correlated with the volume fraction of good solvent *f* and the *m* parameter as depicted in (eq 1).
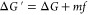
1

Plotting the variation of the degree of aggregation (α) versus the molar fraction of the good solvent affords a sigmoidal curve, characteristic of an isodesmic mechanism, that can be fitted to the SD model to derive the corresponding thermodynamic parameters (Figure 4b,c and Table 1). As expected, the degree of cooperativity σ (defined as the quotient between the elongation, *K*_e_, and the nucleation constants, *K_n_*) is 1, diagnostic of an isodesmic mechanism and a high Gibbs energy release comparable to some other self-assembling systems.^24b,26c,35,36^ The morphology of the aggregates formed upon the supramolecular polymerization of **(*P*)-1** and **(*M*)-1** was visualized by atomic force microscopy (AFM) imaging employing highly oriented pyrolytic graphite (HOPG) as the surface. The AFM images recorded for the samples deposited by spin-coating onto HOPG show the formation of isolated globular aggregates with heights of ∼4 nm (Figures S11, 4d and 4e).

**Table 1:** Thermodynamic Parameters for the Supramolecular Polymerization of **(*P*)-1** and **(*M*)-2**

comp	Δ*G*′^a^	*m*	σ	*K*_e_^b^	*K_n_*^b^
**(*P*)-1**	–36.1 ± 0.8	49.4	1	2.1 × 10^6^	2.1 × 10^6^
**(*M*)-2**	–36.0 ± 0.5	29.5	1.1 × 10^–5^	2.0 × 10^6^	20.4

a In kJ/mol.

b In M^–1^.

**Figure 5:**
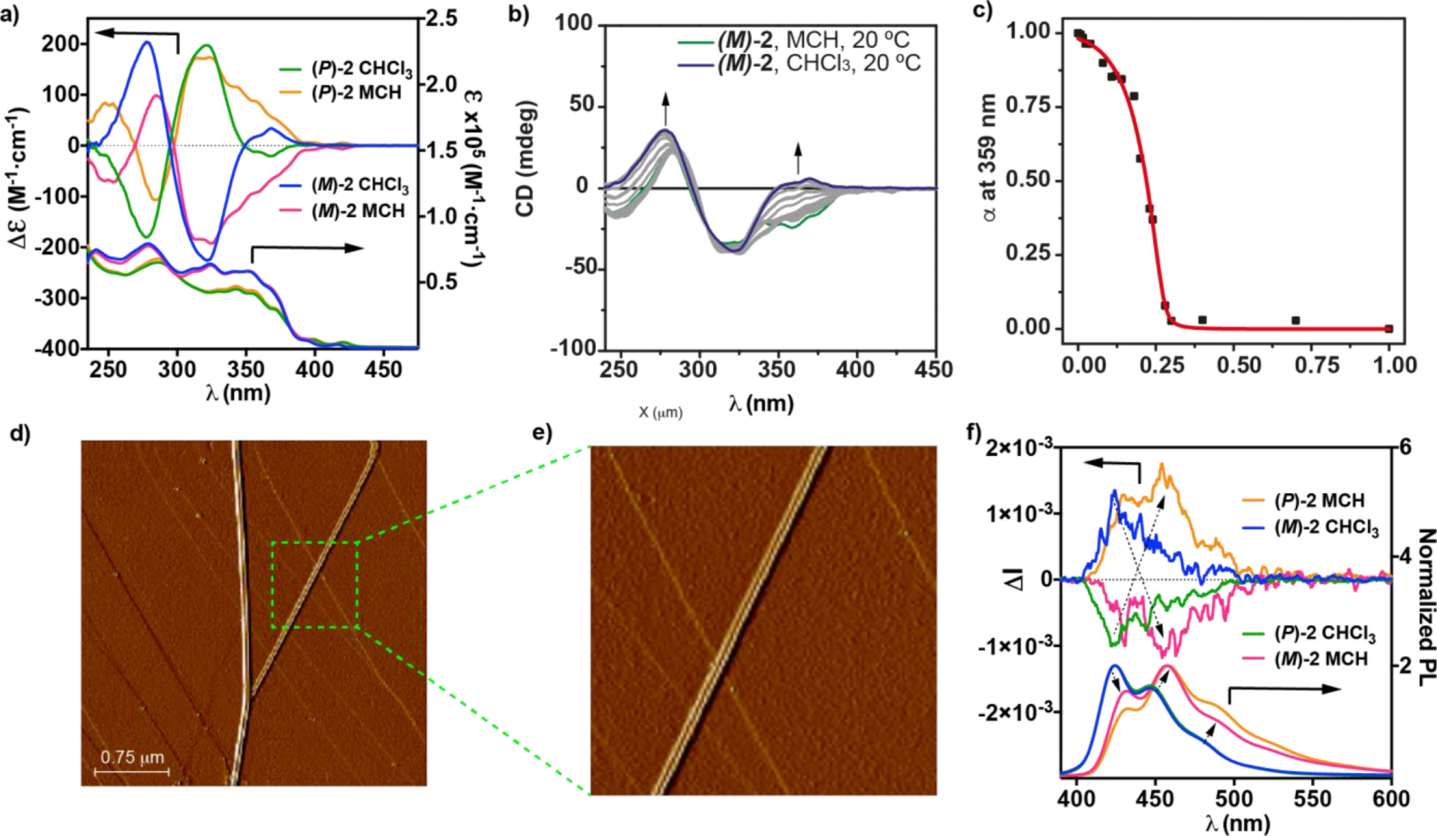
(a) ECD/UV–vis spectra of **(*P*)-2** and **(*M*)-2** in monomeric and aggregated states (CHCl_3_ and MCH, respectively). (b) CD spectra and (c) denaturation curve of **(*M*)-2** in MCH/CHCl_3_ mixtures. The red line in panel (c) depicts the fit to the SD model. (d,e) AFM images of the helical fibers formed upon the supramolecular polymerization of **(*M*)-2** (experimental conditions: HOPG as the surface; *c*_T_ = 10 μM, and MCH as the solvent). (f) CPL/PL spectra of **(*P*)-2** and **(*M*)-2** in monomeric and aggregated states (CHCl_3_ and MCH, respectively) (experimental conditions for UV–vis, ECD, CPL, and PL spectra: *c*_T_ = 10 μM and λ_exc_ = 365 nm; the dashed arrows in (f) depict the changes in the PL and CPL spectra).

Taking into account the changes observed in the UV–vis spectra of **2**, we have also investigated the chiroptical features of the (*P*) and (*M*) enantiomers of this [6]helicene. In CHCl_3_, the ECD spectral pattern mainly displays three Cotton effects at 368, 322, and 278 nm and zero crossing points at 349 and 295 nm (Figures 5a and S9). Remarkably, a strong modification in the ECD pattern is observed in MCH as the solvent. Indeed, the ECD spectra of both **(*P*)-2** and **(*M*)-2** show (i) the low-energy negative vibronic structure band at 368 nm inverts its sign becoming more intense and vibronically structured, (ii) the positive middle-energy band at 322 nm remains with the same sign, and (iii) the monosignate negative band at 278 nm splits into a bisignate band centered at 289 and 250 nm, with a zero-crossing point at 278 nm (Figures 5a and S9). Note that the two very weak low-energy ECD bands at 396 and 416 nm also undergo change in their signs (Figure 5a). The modifications observed in the ECD spectra of **(*P*)-2** and **(*M*)-2** in MCH in comparison to those detected in CHCl_3_ could be indicative of an excitonic coupling of the aromatic units due to the efficient supramolecular polymerization and allows also the utilization of the SD model to unravel the supramolecular polymerization mechanism of this 4,13-substituted helicene. In the case of enantioenriched **(*M*)-2**, plotting the variation of the degree of polymerization α versus the molar fraction of the good solvent yields a clear nonsigmoidal curve that implies a cooperative supramolecular polymerization mechanism (Figure 5b,c). This mechanism contrasts with the isodesmic mechanism shown by **(*P*)-1**. Fitting the nonsigmoidal curve obtained in the denaturation experiment performed with **(*M*)-2** yields the thermodynamic parameters collected in Table 1. **(*M*)-2** presents a similar Gibbs energy release to **(*P*)-1**, but the degree of cooperativity is higher than that derived for [5]helicenes,^26c^ atropisomers,^25^ or planar self-assembling units.^35,36^

To visualize the morphology of the aggregates formed upon the supramolecular polymerization of the racemic mixture of **(*M*)-2** and **(*P*)-2**, we have registered AFM images of a spin-coated solution of this mixture onto HOPG. Unlike [6]helicenes **1**, which form globular nanoparticles (Figure 4d), the AFM images of [6]helicenes **2** show the presence of well-defined fibrillar aggregates with helical character (Figure S12). This helical character is also visualized in the AFM images of the enantioenriched samples of **(*M*)-2** and **(*P*)-2**. The AFM images of these enantioenriched samples present one-dimensional fibrillar aggregates of several micrometers length and a typical height of around 2.5 nm (Figures 5d,e and S13 and S14). Delightfully, a closer inspection of the AFM images of **(*M*)-2** shows the helical morphology of these fibrillar aggregates that bundle into thicker fibers. Similar findings have been visualized for the enantioenriched **(*P*)-2** (Figure S15).

The emission properties of both enantiomers of the 2,15- and 4,13-substituted [6]helicenes **1** and **2** were also recorded. Fluorescence spectra of **(*P*)-1** and **(*M*)-1** in CHCl_3_ show classical vibronic structured photoluminescence (PL) previously reported for [6]helicenes^9a,37^ with three consecutive maxima at 430, 455, and 486 nm (λ_exc_ = 365 nm, ϕ = 0.35, and τ = 8.5 ns) that also appear in the corresponding CPL spectra with a remarkable dissymmetry factor—defined as *g*_lum_ = 2(*I*_L_ – *I*_R_)/(*I*_L_ + *I*_R_), *I*_L_ and *I*_R_ being the left- and right-handed luminescence emissions, respectively—with maximum *g*_lum_ values of +2.3/–2.6 × 10^–2^ (λ_exc_ = 365 nm) for **(*P*)-1** and **(*M*)-1**, respectively (Figure 4f). In agreement with the minute changes observed in the corresponding UV–vis and ECD spectra (Figures 2a and 4a) of **(*P*)-1** and **(*M*)-1** in MCH and CHCl_3_, the PL spectra of these enantioenriched 2,15-substituted [6]helicenes in MCH show a slight red shift compared with the CHCl_3_ solution, with maxima at 436, 462, and 493 (λ_exc_ = 365 nm, ϕ = 0.59, and τ =12.2 ns) also present in the CPL spectra with maximum *g*_lum_ values of +2.0/–2.3 × 10^–2^ (λ_exc_ = 365 nm), respectively. For comparison, maximum absorption dissymmetry factors *g*_abs_ of 1.6 × 10^–2^ and 1.1 × 10^–2^ in CHCl_3_ and MCH are observed respectively for **(*P*)-1** at 422 and 427 nm, respectively (see Figure S10a). Overall, the nonpolarized and polarized absorption and emission characteristics of 2,15-substituted **(*P*)-1** and **(*M*)-1** in the aggregated and nonaggregated states are of similar shapes and magnitude, with notably strong dissymmetry factors conserved within the supramolecular assemblies.

Regarding **(*P*)-2** and **(*M*)-2**, their fluorescence spectra in CHCl_3_ display the classical vibronic structured luminescence (λ_exc_ = 365 nm, ϕ = 0.52, and τ = 7.3 ns) of [6]helicenes^37^ with three consecutive maxima at 424, 446, and 478 nm (Figure 5f). In MCH, the fluorescence spectra of **(*P*)-2** and **(*M*)-2** show a slight red shift, with maxima at 433, 458, and 490 nm (λ_exc_ = 365 nm, ϕ = 0.36, and τ = 2.25 ns) (Figure 5f). Satisfactorily, the CPL response of **(*P*)-2** exhibits a sign inversion from negative to positive upon aggregation, in full agreement with the sign inversion of the low-energy ECD-active bands. The corresponding CPL spectra display max *g*_lum_ values of −1.1/+1.3 × 10^–3^ (Figure 5f) in CHCl_3_, while they show a small increase and inversion in MCH, i.e., of +1.4/–1.2 × 10^–3^, for the (*P*) and (*M*) enantiomers, respectively. For comparison, maximum absorption dissymmetry factors *g*_abs_ of 3.2 × 10^–3^ and 4 × 10^–3^ in CHCl_3_ and MCH were obtained for **(*P*)-2** at 318 and 324 nm, respectively (see Figure S10b). To our knowledge, this is the first observation of CPL sign inversion upon assembly in helicenes.^26a,28,38^ The sign of these low-energy ECD active bands in helicene derivatives is known to be highly substituent-sensitive^39^ and we now demonstrate that it is also impacted by the self-assembly, while the inherent chirality of the helical core is not changed.

In summary, our synthetic strategy appeared efficient to obtain robust chiral supramolecular assemblies from monomers consisting of a central helical core and strongly aggregating achiral bisamide substituents. The obtained supramolecular aggregates appear highly stable both in solution and on surfaces, with strong and processable chiroptical activity. Based on these characteristics and according to the literature on helicenes and on helical supramolecular assemblies displaying strong spin selectivity,^11,16b,27b^ it appeared appealing to examine how effective these helicene-based helical SPs were as spin filters.

### Spin Selectivity of Helicene-Based Supramolecular Assemblies

**Figure 6:**
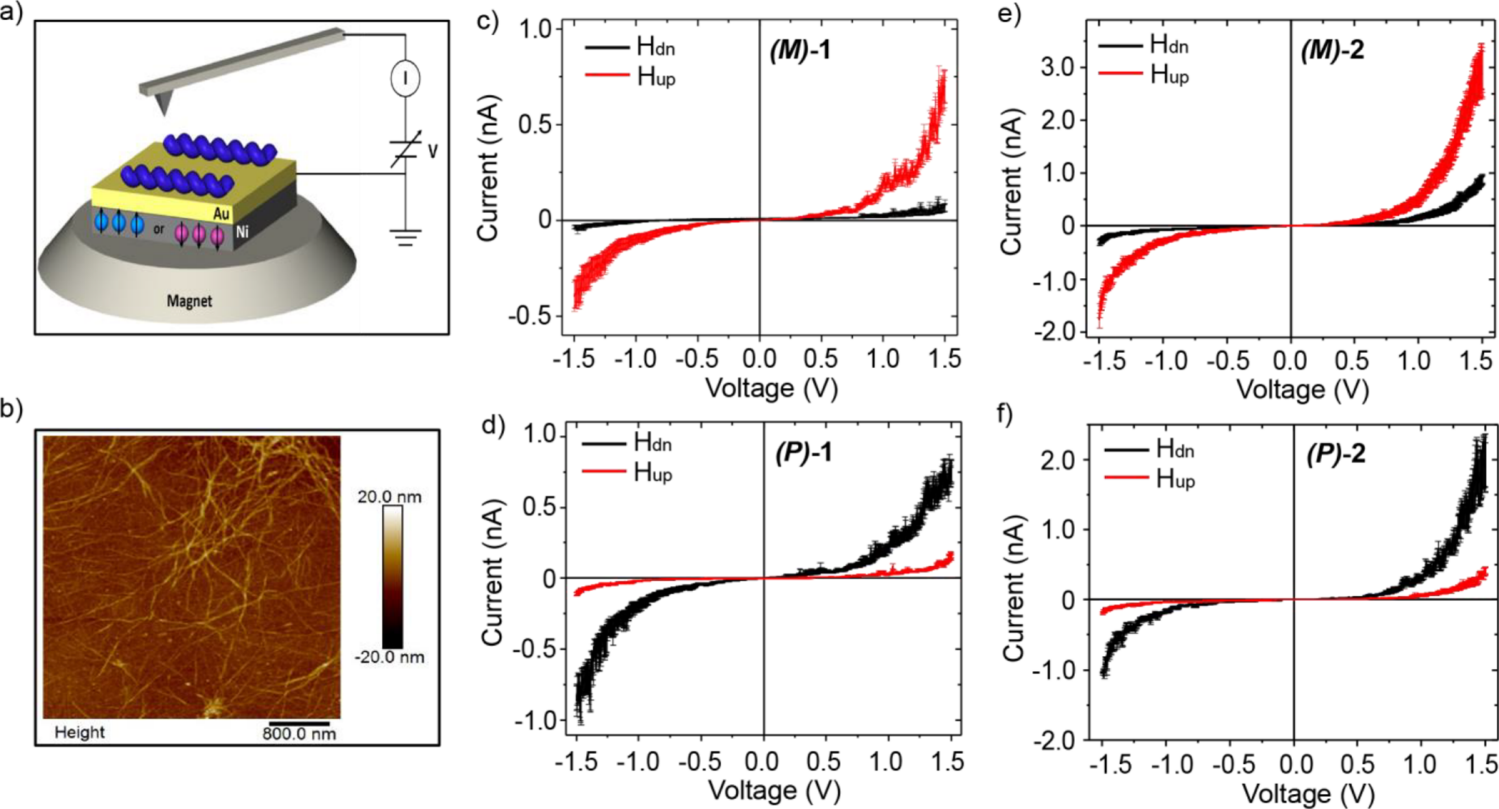
Spin-dependent transport properties measured with mc-AFM. (a) mc-AFM setup in which the molecules are deposited on a ferromagnetic substrate and the conduction between the substrate and the AFM tip, through the molecules, is measured for the substrate magnetized with its north pole up or down relative to the molecular layer. (b) AFM image of the substrate on which the **(*P*)-2** molecules were deposited. Panels (c) and (d) present the averaged current versus voltage (*I*–*V*) curves recorded for **(*M*)-1** and **(*P*)-1** samples, respectively, with the magnet north pole pointing down (black) or up (red). Panels (e) and (f) presents the averaged *I*–*V* curves recorded for **(*M*)-2** and **(*P*)-2** samples.

Another appealing feature of organized helical molecules is their ability to generate electron spin polarization, a behavior that is intensively targeted for spintronic applications.^6^ Magnetic conducting atomic force microscopy^11a,40^ (mc-AFM) measurements were thus performed to investigate the spin selectivity of the electron transport through a layer of the helicene-based polymer. For this purpose, the helicene samples were deposited on a gold-coated nickel substrate (Ni 100 nm and Au 8 nm), which can be magnetized with the north magnetic pole pointing toward the layer (up) or away from the layer (down) (Figure 6a), using an external magnetic field. The nonmagnetic AFM tip was grounded, while the potential on the Au/Ni surface was varied. Prior to the current versus voltage (*I*–*V*) studies, the morphology of the samples was analyzed using AFM topography images. Figure 6b presents an image of the surface on which the (*P*)-**2** molecules were deposited. The images for surfaces covered with the (*P*)-**1** and (*M*)-**1** molecules are shown in the Supplementary Information (Figure S16). Similar to the case of HOPG surfaces (vide supra), the figures clearly indicate that the (*P*)-**1** and (*M*)-**1** samples form globular aggregates, while the (*P*)-**2** and (*M*)-**2** samples form helical nanofibers.

Figure 6c,d shows the average *I–V* curves of the **(*M*)-1** and **(*P*)-1** samples, while Figure 6e,f presents the averaged *I–V* curves of the **(*M*)-2** and **(*P*)-2** samples, respectively. The molecules were deposited on the substrate by drop casting. All samples show clear dependence of the current on the direction of the magnetization of the substrate. Each curve is an average over at least 50 individual measurements (see Figures S17 and S18 in the Supporting Information). The applied magnetic field controls the spin selectivity in the case of the *M* enantiomers in an opposite manner to that of the *P* enantiomer, which advocates the role of chirality in spin-dependent transport properties. These results are in excellent agreement with previous reports^11a,40^ in which the spin selectivity reverses when the chirality changes. Moreover, a nonlinear dependence of the current on the voltage curves was observed and the currents corresponding to each of the spins start at a different voltage, suggesting the presence of a different barrier to the injection of the individual spins.

**Figure 7:**
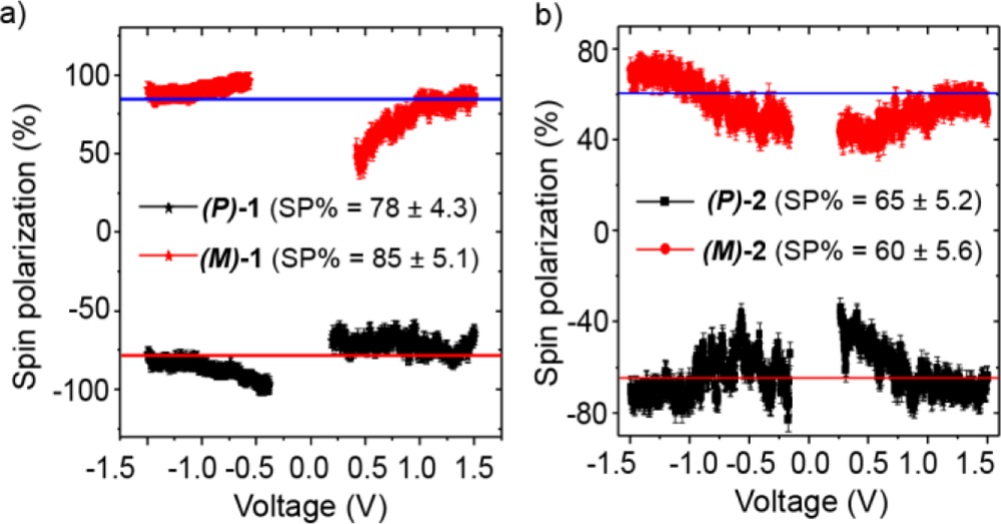
Spin polarization percentage (SP%) as a function of applied bias for (a) **(*P*)-1** and **(*M*)-1** and (b) **(*P*)-2** and **(*M*)-2** samples, respectively. SP% =  × 100.

Furthermore, the percentage of spin polarization (SP%) is calculated^11a,40^ when SP% =  × 100, where I_up_ and I_dn_ represent the current when the north pole of the magnetic field is directed upward or downward direction, respectively. The dependences of the spin polarization on the applied voltage are shown in Figure 7a,b and are 80% ± 5 and 60% ± 5% for **(*P*)-1** and **(*M*)-1** and **(*P*)-2** and **(*M*)-2** enantiomers, respectively. The observed values of SP% are high, while the molecules that form globular aggregates, **(*P*)-1** and **(*M*)-1**, show somewhat higher values than those forming helical wires **(*P*)-2** and **(*M*)-2**. Overall, these values for supramolecular assemblies of helicenes are found to be much higher than those of self-assembled monolayers (SP ∼ 6–40%).^11^ The difference may result from the higher polarizability of the supramolecular structures.^41,42^ Indeed, it is now known that there is correlation between chiroptical activity and spin polarization.^16b,42^ Thus, the stronger chiroptical activity of system **1** over **2** (i.e., stronger ECD responses at low energy) may account for its higher spin filtering effects. Indeed, ECD spectra of helicene films over the quartz substrate show stronger low-energy response for **(*P*)-1** and **(*M*)-1** than for **(*P*)-2** and **(*M*)-2** (see Figure S19a and b). It is important to appreciate that 80 and 60% spin polarization (Figure 7) means ratios of 10:1 and 5:1 between the current with the preferred spin to that of the unpreferred spin. In other words, when the magnetic field is up, the *M* enantiomers selectively let the electron spin pass through the chiral layer, and vice versa, thus relating the absolute configuration of the helicene to the spin polarization. Clearly, the results indicate that the supramolecular structure affects the spin polarization and it does not depend solely on the structure of the monomer.

**Figure 8:**
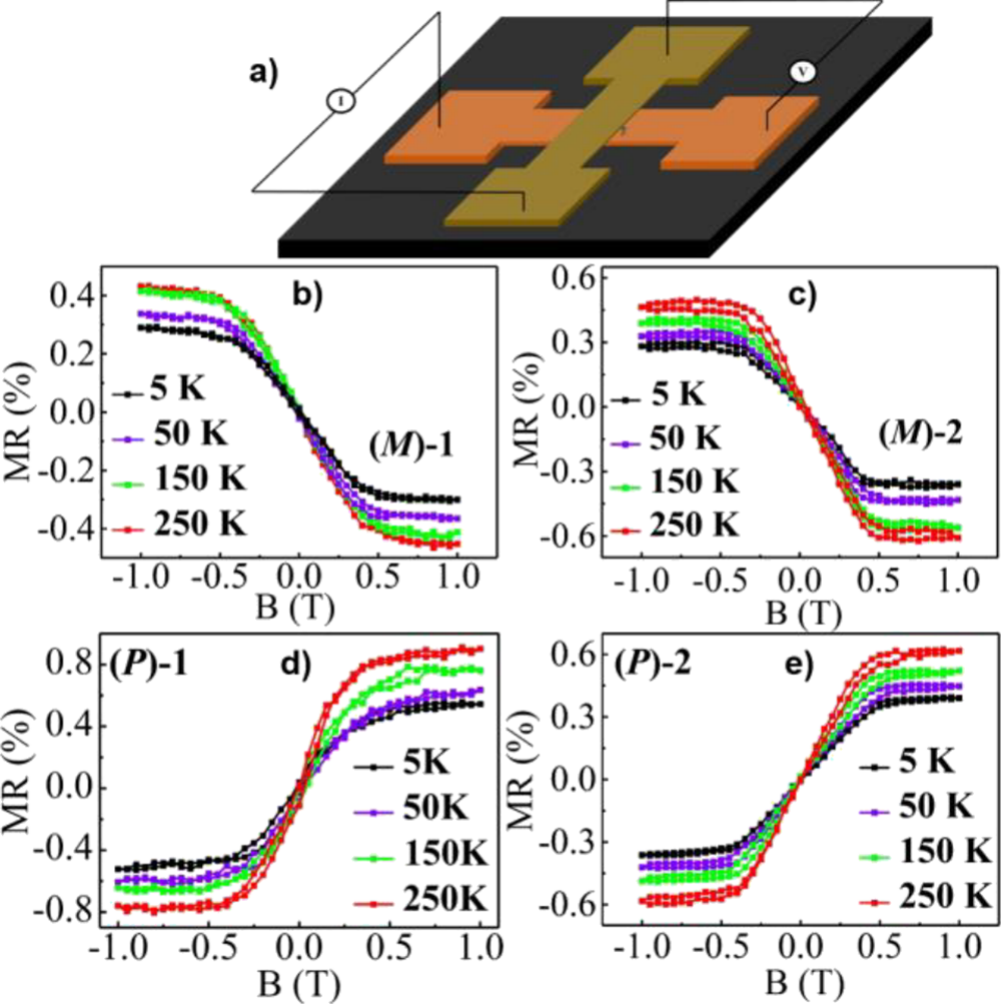
Magnetoresistance results. (a) Schematic of the four-probe magnetoresistance (MR) device with gold (Au) as the bottom electrode and Ni as the top electrode. Temperature-dependent magnetoresistance (MR) obtained for (b) and (c) **(*M*)-1** and **(*M*)-2**. Panels (d) and (e) present MR of **(*P*)-1** and **(*P*)-2** respectively at different temperatures as a function of the magnetic field with an input current of 0.1 mA.

For establishing the properties of the molecules as spintronic elements, magnetoresistance measurements were also performed (Figure 8). A crossbar configuration was used for the magnetoresistance (MR) device that was produced as described in ref (11a) (Figure 8a). Molecules were spin coated on the top of the bottom electrode. On the top of the polymer film, an insulating buffer layer of 1.5 nm magnesium oxide (MgO) was grown by e-beam evaporation followed by Ni and Au having thicknesses of 40 and 20 nm, respectively, using a shadow mask with a line width of ∼20 μm (see the experimental details about the set-up in the Supporting Information).

The current through the chiral molecules was studied when the magnetic field was varied. Figure 8b,c shows the MR of **(*M*)-1** and **(*M*)-2**, while the MR values of **(*P*)-1** and **(*P*)-2** are presented in Figure 8d,e, respectively, measured at different temperatures with a constant input current of 0.1 mA. The MR (%) is defined as MR (%) =  × 100, where *R*(*B*) and *R*(0) are the resistances measured at the magnetic field up to 1 T and zero-magnetic field, respectively. Note that here we find a small value of MR (%) due to the large number of pinholes. Furthermore, due to this issue, no efficient devices were obtained for **(*M*)-1**–**2** and **(*P*)-1**–**2**. However, the signal-to-noise ratio is excellent and it is evident that the MR curves are asymmetric with respect to the magnetic field and that the asymmetry depends on the handedness of the molecules. These results are consistent with the mc-AFM data (Figure 6). The value of MR (%) increases with the temperature, which confirms the increase of spin polarization with the temperature, probably due to the role of phonon-enhanced spin–orbit coupling.^41,42^

## Conclusions

In conclusion, we have demonstrated the efficient formation of supramolecular polymers based on a carbo[6]helicene scaffold, whose racemic mixture self-assembles as a conglomerate, and the deep influence of the substituent location in the helical backbone on the polymerization mechanisms and chiroptical properties. On the one hand, the 2,15-substituted derivative **1** experiences an isodesmic supramolecular polymerization mechanism, generating globular nanoparticles that maintain CPL with *g*_lum_ values as high as 2 × 10^–2^. On the other hand, the 4,13-substituted derivative **2** follows a cooperative supramolecular polymerization mechanism generating helical one-dimensional fibers. Remarkably, [6]helicenes **2** exhibit a unique modification in their ECD spectral pattern showing sign inversion of low-energy bands. In parallel, the CPL response shows a sign inversion with *g*_lum_ values of 1.2 × 10^–3^, representing the first example of a CPL switch upon supramolecular polymerization. Both molecules, when assembled on a surface, are excellent electron spin filters at room temperature. This directly results from two main features: (i) strong ability of these helicene derivatives to self-assemble onto a (conductive) surface and (ii) strong chiroptical activity. The spin filtering indicates that the current of electrons with the preferred spin is more than four times larger than the current of the electrons with unpreferred spin. This work highlights the potential of helicenes as appealing building blocks in the field of CPL-active supramolecular polymers and spintronics, paving the way to new chiral materials with enhanced properties and applications, efforts in that direction being currently ongoing in our laboratories.

## Supporting Information Available

Full experimental details, characterization and additional spectroscopic measurements, additional AFM images, additional details on mc-AFM, solid-state measurements, and supporting references including Figures S1–S20 (PDF).

## Abbreviations

SP: supramolecular polymerization
ECD: electronic circular dichroism
CPL: circular polarized luminescence
ROESY: rotating frame Overhauser effect spectroscopy
MCH: methylcyclohexane
VT: variable temperature
AFM: atomic force microscopy
