# Supplementary material for: Mutual Monomer Orientation To Bias the Supramolecular Polymerization of [6]Helicenes and the Resulting Circularly Polarized Light and Spin Filtering Properties
Source: J Am Chem Soc. 2022 Apr 11;144(17):7709–19. doi: 10.1021/jacs.2c00556 (PMC9073930; doi:10.1021/jacs.2c00556)
Supplement: Supplementary file 1 — ja2c00556_si_001.pdf [file ja2c00556_si_001.pdf]

Mutual Monomer Orientation To Bias the Supramolecular Polymerization of [6]Helicenes and the Resulting Circularly Polarized Light and Spin Filtering Properties.

Rafael Rodríguez,[a,‡] Cristina Naranjo,[b,‡] Anil Kumar,[c] Paola Matozzo,[a] Tapan Kumar Das,[c] Qirong Zhu,[c] Nicolas Vanthuyne,[d] Rafael Gómez,[b] Ron Naaman,*[,c] Luis Sánchez,*[,b] Jeanne Crassous*[,a]

[a] Univ Rennes, CNRS, ISCR (Institut des Sciences Chimiques de Rennes) – UMR 6226, F-35000 Rennes, France

[b] Departamento de Química Orgánica, Facultad de Ciencias Químicas, Universidad Complutense de Madrid, 28040 Madrid, Spain

[c] Department of Chemical and Biological Physics, Weizmann Institute of Science, Rehovot 76100, Israel

[d] Aix Marseille Université, Centrale Marseille, CNRS, iSm2, UMR 7313, Marseille, France

Contents:

Experimental section S-2

*Synthesis and HPLC separation of derivatives **1** and **2*** S-3

*NMR experiments of derivative **1*** S-10

*NMR experiments of derivative **2*** S-13

Additional spectroscopic studies S-16

Additional AFM experiments S17

Sample preparation for magnetic- conductive atomic force microscopy (mc-AFM) S18

*Spin dependent transport properties for **(M)-1** and **(P)-1** enantiomers* S-21

*Spin dependent transport properties for **(M)-2** and **(P)-2** enantiomers* S-22

Magnetoresistance measurements S-22

Solid State ECD and UV-Vis studies S-23

Racemization tests S-23

Supporting References S-24

1. Experimental section

General.

All solvents were dried according to standard procedures.

Reagents were used as purchased from Sigma-Aldrich, Alfa Aesar, Fluorochem or TCI Europe without further purification.

All air-sensitive reactions were carried out under argon atmosphere.

Flash chromatography was performed using silica gel (Merck, Kieselgel 60, 230-240 mesh or Scharlau 60, 230-240 mesh).

Analytical thin layer chromatography (TLC) was performed using aluminum-coated Merck Kieselgel 60 F254 plates.

NMR spectra were recorded on a Bruker Avance 400 (^{1}H: 400 MHz; ^{13}C: 100 MHz) spectrometer at 298 K using partially deuterated solvents as internal standards. Coupling constants (*J*) are denoted in Hz and chemical shifts (δ) in ppm. Multiplicities are denoted as follows: s = singlet, d = doublet, t = triplet, m = multiplet, q = quadruplet, br = broad.

High-resolution mass spectra (HR-MS) determinations were performed at CRMPO on a Bruker MaXis 4G and Ultraflex III by ASAP (+ or -) or ESI techniques with CH_2Cl_2 as solvent. Experimental and calculated masses are given with consideration of the mass of the electron.

FT-IR spectra were recorded on a Bruker Tensor 27 (ATR device) spectrometer. UV-Vis spectra were recorded on a Varian Cary 50 spectrophotometer.

High resolution mass spectra (HRMS) were recorded on a FTMS Bruker APEX Q IV spectrometer. FTIR spectra were recorded on a JASCO-FT-IR-6800 spectrometer using a CaF_2 cell with a path length of 0.1 mm. UV-Vis spectra were recorded on a JASCO V-630/750 spectrophotometer by using quartz cuvettes (Hellma). Thermal experiments were performed at constant cooling rates of 1 K min^{-1} from 283 to 363 K in methylcyclohexane (MCH). Circular dichroism (CD) measurements were performed on a JASCO-1500 dichrograph equipped with a Peltier thermoelectric temperature controller. Atomic force microscopy (AFM) was performed on a SPM Nanoscope IIIa multimode microscope working on tapping mode with a RTESPA tip (Veeco) at a working frequency of ~235 kHz.

Circular dichroism (CD) measurements were performed on a Jasco J-815 Circular Dichroism Spectrometer IFR140 facility. Part of this work has been performed using the PRISM core facility (Biogenouest©, UMS Biosit, Université de Rennes 1 - Campus de Villejean-35043 Rennes Cedex, France).

GPC purification was performed in a Japan Analitical Instrument eluting in $CHCl_3$ at 10 ml/min flow.

Circularly polarized luminescence (CPL) measurements were performed using an in-house-developed JASCO CPL spectrofluoropolarimeter. The samples were excited using a 90°-geometry with a green InGaN (3 mm, 2 V) LED source (Luckylight Electronics Co., LTD, λ_{max} = 517 nm, HWHM = 15 nm). The following parameters were used: emission slit width ≈ 10 nm, integration time = 4 sec, scan speed = 50 nm/min, 3 accumulations.

Optical Rotation measurements were performed on a Jasco P-2000 polarimeter.

The thermodynamic parameters associated to the supramolecular polymerization mechanisms were derived by applying the solvent denaturation model published by Meijer and coworkers.[S1]

Fluorescence spectra were recorded on a FL 920 Edinburgh fluorimeter. Fluorescence quantum yields Φ were measured in diluted solution using the following equation:

$$\Phi_X = \Phi_{ST}\left(\frac{Grad_X}{Grad_{ST}}\right)\left(\frac{\eta_X^2}{\eta_{ST}^2}\right)$$

Where the subscripts ST and X denote standard and sample respectively, Φ is the fluorescence quantum yield, Grad is the gradient from the plot of integrated fluorescence intensity vs absorbance, and η the refractive index of the solvent. Reference for fluorescence quantum yields used herein are quinine sulfate in 0.5 M sulfuric acid (Excitation of reference and sample compounds was performed at the same wavelength).

The decays were calculated by using an Ocean optics QEPro CCD detector (Range: 350–1100 nm). The excitation source has been a picosecond laser diode (10 KHz to 100 MHz) at 375 nm capable of operating in Burst mode below 10 KHz. For the detection windows, signals in UV-Vis (350–950 nm) are recorded as a function of time over a range of 135 nm simultaneously using a Hamamatsu C10910-25 streak camera mounted with a slow single sweep drawer. The range of measurable lifetimes is 100–300 μs.

2. Synthetic details

4,13-Diethynyl-[6]helicene, 2,15-Diethynyl-[6]helicene, 3,4,5-tris(dodecyloxy)-*N*-(4-iodophenyl)benzamide and 3,4,5-tris(dodecyloxy)-*N*-(2-(4-iodobenzamido)ethyl)benzamide were prepared according to previously reported synthetic procedures and showed identical spectroscopical properties to those reported therein.[S2-S5]

General Protocol for Sonogashira Coupling

The corresponding bis-ethynyl-[6]helicene and aryl iodide were dissolved in a mixture of dry THF and Et_3N (1/1) and was degassed by 3 Vacuum/Ar cycles. CuI and $Pd(PPh_3)_4$ were added and the reaction mixture was stirred for 2 hours at 50ºC. The organic layer was evaporated under reduced pressure and the residue was purified by column chromatography (silica gel) using heptane/ethyl acetate (1/1) for the ethylene diamide derivatives **1** and **2**. The fractions containing the product were evaporated under reduced pressure and the residue was purified by recycling GPC eluting with $CHCl_3$, affording the corresponding the pure compounds.

2,15-[6]Helicene derivative (1)

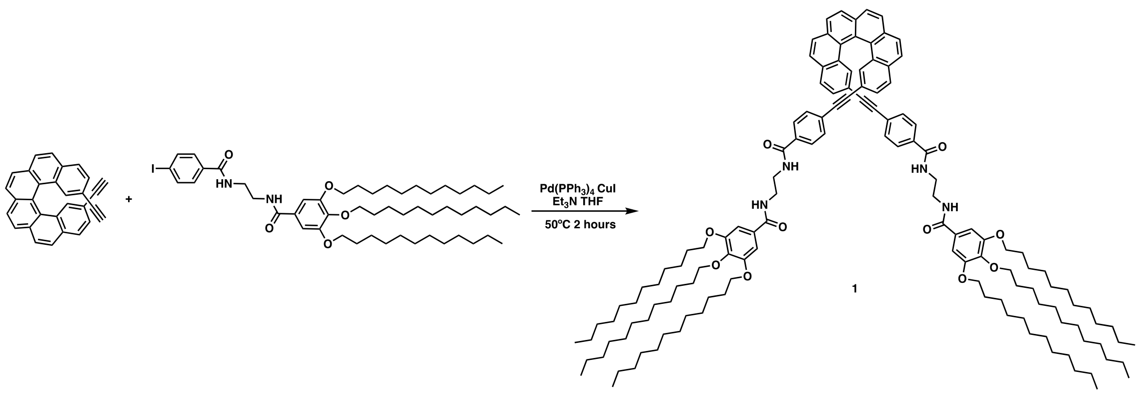

Following the general protocol for Sonogashira cross-coupling, *rac*-[6]helicene (10 mg, 0.027 mmol, 1 eq.), 3,4,5-tris(dodecyloxy)-*N*-(2-(4-iodobenzamido)ethyl)benzamide (64 mg, 0.067 mmol, 2.5 eq.), $Pd(PPh_3)_4$ (4 mg, 0.0035mmol, 0.05 eq.) and CuI (1 mg, 0.0067 mmol, 0.1 eq.) were dissolved in 4 mL solution of THF/Et_3N (1/1). After the purification following the general protocol depicted above, **1** was obtained as a light-yellow solid (47 mg, 67 %).

^{1}H NMR (CD_2Cl_2, 400 MHz, δ ppm): 8.05–7.95 (m, 7H), 7.91 (d, *J* = 8.6 Hz, 2H), 7.82–7.79 (m, 5H), 7.76 (d, *J* = 8.3 Hz, 2H), 7.53 (q, *J* = 5.6 Hz, 2H), 7.42–7.38 (m, 2H), 7.34 (dd, *J* = 8.2, 5.3 Hz, 5H), 7.08 (s, 4H), 4.01 (t, *J* = 6.5 Hz, 13H), 3.71 (s, 8H), 1.84–1.72 (m, 12H), 1.47 (tt, *J* = 9.8, 5.1 Hz, 12H), 1.37–1.19 (m, 98H), 0.88 (q, *J* = 6.7 Hz, 18H). See assignments in Figure S1.

^{13}C NMR (CD_2Cl_2, 101 MHz, δ ppm): 168.9, 168.0, 153.13, 153.07, 141.2, 133.4, 132.8, 132.2, 131.82, 131.77, 131.5, 129.1, 128.8, 128.0, 127.6, 127.5, 127.5, 127.4, 127.1, 127.0, 127.0, 123.9, 118.9, 92.4, 87.87, 77.2, 73.5, 69.2, 41.1, 31.9, 31.9, 30.4, 29.8, 29.7, 29.8, 29.7, 29.7, 29.5, 29.6, 29.4, 26.1, 26.1, 22.7, 22.7, 14.1.

HRMS (MALDI-TOF) calcd. for $C_{134}H_{188}N_4O_{10}$ $[M+H]^+$, 2014.4398; found, 2014.4374.

Experimental optical rotation values: ***P*-(+)-1**: [α] = +1500 (CH_2Cl_2, 9.6 mg·L^{-1}), ***M*-(-)-1**: [α] = -1502 (CH_2Cl_2, 6 mg·L^{-1}).

Chiral HPLC separation for compound 1

• The sample is dissolved in dichloromethane, injected on the chiral column, and detected with an UV detector at 254 nm, a circular dichroism detector at 254 nm and a polarimetric detector. The flow-rate is 1 mL/min.

Column	Mobile Phase	t1	k1	t2	k2	α	Rs
**Chiralpak IF**	Heptane / 2-PrOH / dichloromethane (70/20/10)	5.10	0.73	6.52	1.21	1.66	2.65

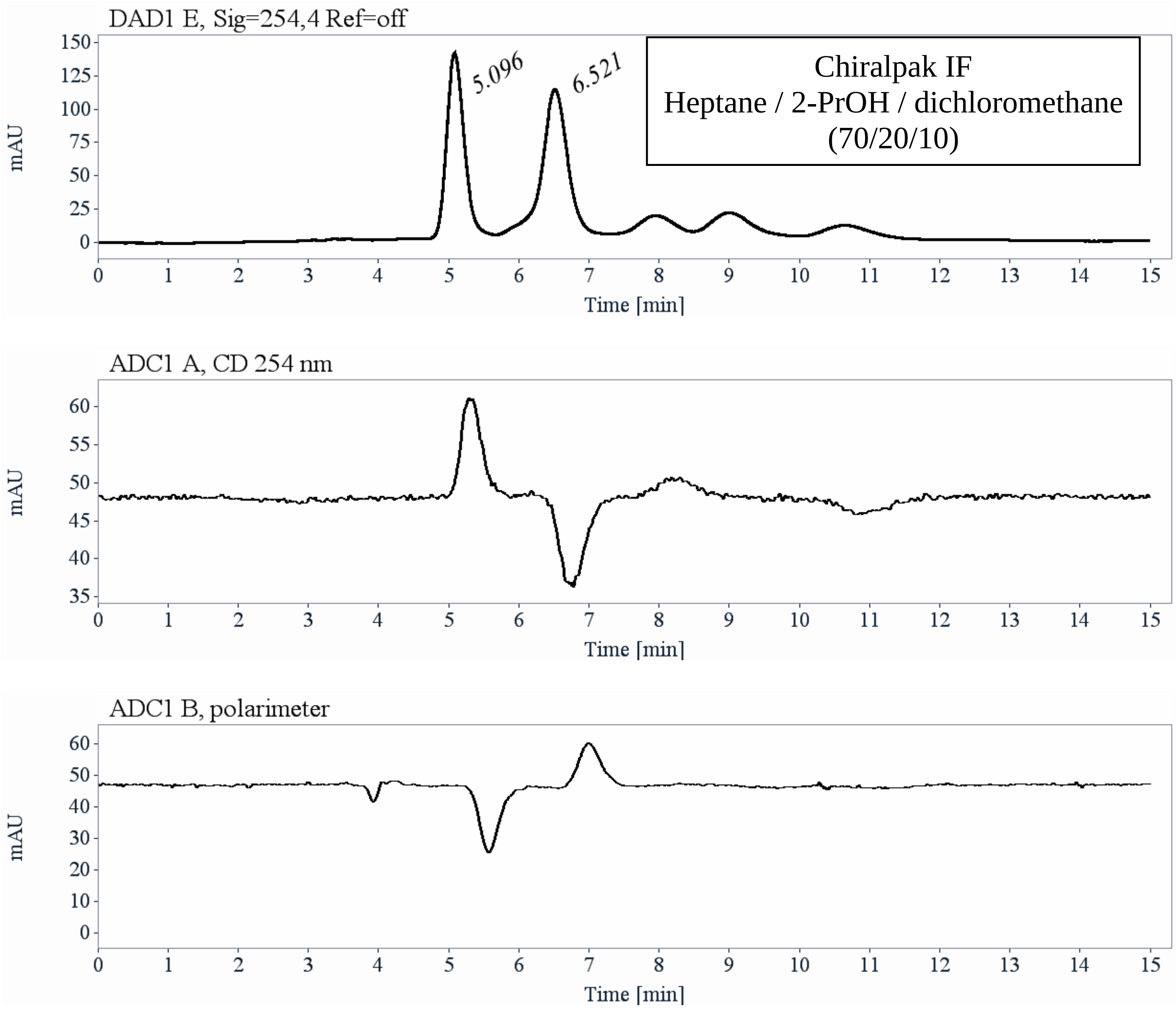

RT [min]	Area	Area%	Capacity Factor	Enantioselectivity	Resolution (USP)
5.10	2435	46.18	0.73		
6.52	2838	53.82	1.21	1.66	2.65
Sum	5273	100.00			

Preparative separation for compound **1**:
• Sample preparation: About 29 mg of compound **1** are dissolved in 7 mL of a mixture dichloromethane and ethanol (85/15).
• Chromatographic conditions: Chiralpak IF (250 x 10 mm), hexane / 2-PrOH / dichloromethane (70/20/10) as mobile phase, flow-rate = 5 mL/min, UV detection at 254 nm.
• Injections (stacked): 100 times 70 μL, every 9.2 minutes.

• First fraction: 7.9 mg of the first eluted with ee > 99.5 %

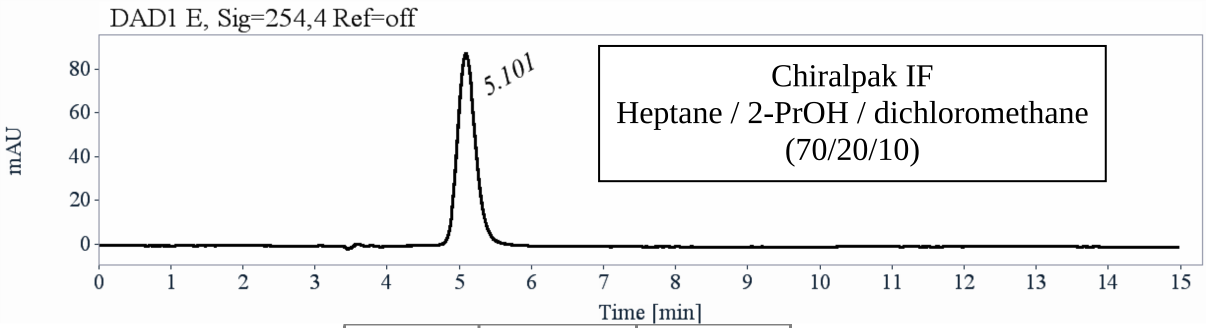

RT [min]	Area	Area%
5.10	1589	100.00
Sum	1589	100.00

• Second fraction: 10 mg of the second eluted with ee > 97.5 %

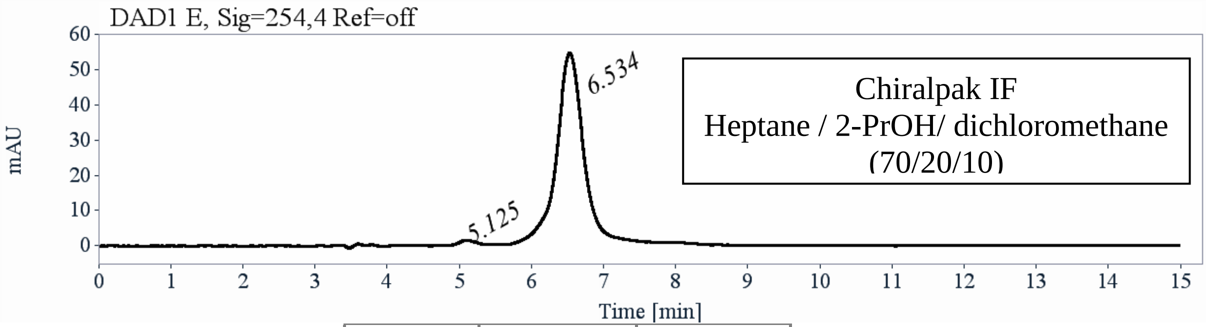

RT [min]	Area	Area%
5.13	17	1.16
6.53	1414	98.84
Sum	1430	100.00

See Figure S20 for configurational stability.

4,13-[6]Helicene derivative (2)

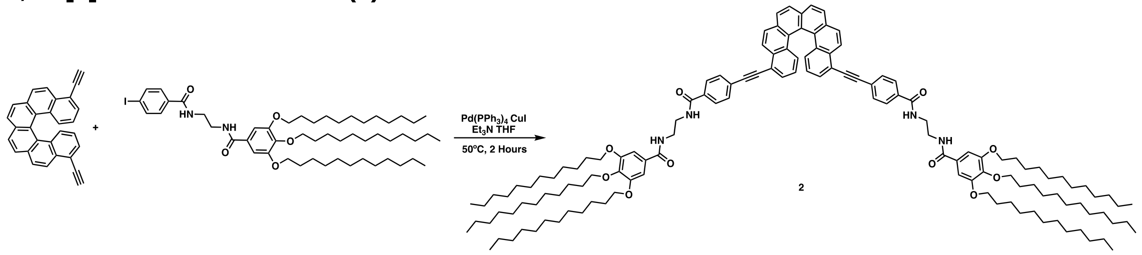

Following the general protocol for Sonogashira cross-coupling, *rac*-[6]helicene (15 mg, 0.04 mmol, 1 eq.), 3,4,5-tris(dodecyloxy)-*N*-(2-(4-iodobenzamido)ethyl)benzamide (113 mg, 0.12 mmol, 3 eq.), $Pd(PPh_3)_4$ (4 mg, 0.002 mmol, 0.05 eq.) and CuI (1 mg, 0.011 mmol, 0.18 eq.) were dissolved in 4 mL solution of THF/Et_3N (1/1). After the purification following the general protocol depicted above, **2** was obtained as a light-yellow solid (26 mg, 33 %).

^{1}H NMR (CD_2Cl_2, 400 MHz, δ ppm): 8.59 (d, J = 8.7 Hz, 2H), 8.13–8.00 (m, 6H), 7.95–7.87 (m, 4H), 7.79–7.71 (m, 4H), 7.63 (d, J = 8.6 Hz, 2H), 7.52 (dd, J = 7.2, 1.1 Hz, 2H), 7.40 (t, J = 4.8 Hz, 2H), 7.18 (t, J = 4.8 Hz, 2H), 7.06 (s, 4H), 6.71 (dd, J = 8.6, 7.2 Hz, 2H), 4.03 (dt, J = 10.0, 6.5 Hz, 12H), 3.75 (q, J = 5.2 Hz, 8H), 1.89–1.72 (m, 12H), 1.54–1.44 (m, 12H), 1.38–1.21 (m, 97H), 0.88 (q, J = 6.8 Hz, 18H). See assignments in Figure S7.

^{13}C NMR (CD_2Cl_2, 100 MHz, δ ppm): 169.0, 168.1, 153.3, 141.3, 133.6, 133.4, 132.0, 131.9, 131.5, 130.5, 129.9, 129.0, 128.9, 128.1, 127.6, 127.4, 127.1, 125.8, 124.4, 124.2, 120.0, 105.7, 93.4, 90.9, 77.4, 73.7, 69.4, 53.6, 41.3, 41.2, 32.1, 29.9, 29.9, 29.8, 29.8, 29.7, 29.5, 29.5, 29.5, 26.3, 26.2, 22.8, 22.8, 14.3.

HRMS (MALDI-TOF) calcd. for $C_{134}H_{188}N_4O_{10}$ $[M+H]^+$, 2014.43982; found, 2014.4377.

Experimental optical rotation values: ***P*-(+)-2**: [α] = +1967 (CH_2Cl_2, 9 mg·mL^{-1}), ***M*-(-)-2**: [α] = -1912 (CH_2Cl_2, 9.6 mg·mL^{-1}).

Analytical chiral HPLC separation

• The sample is dissolved in dichloromethane, injected on the chiral column, and detected with an UV detector at 254 nm and a circular dichroism detector at 254 nm. The flow-rate is 1 mL/min.

Column	Mobile Phase	t1	k1	t2	k2	α	Rs
**Chiralpak IF**	Heptane / 2-PrOH / dichloromethane (70/20/10)	7.24 (+)	1.45	11.17 (-)	2.79	1.92	4.44

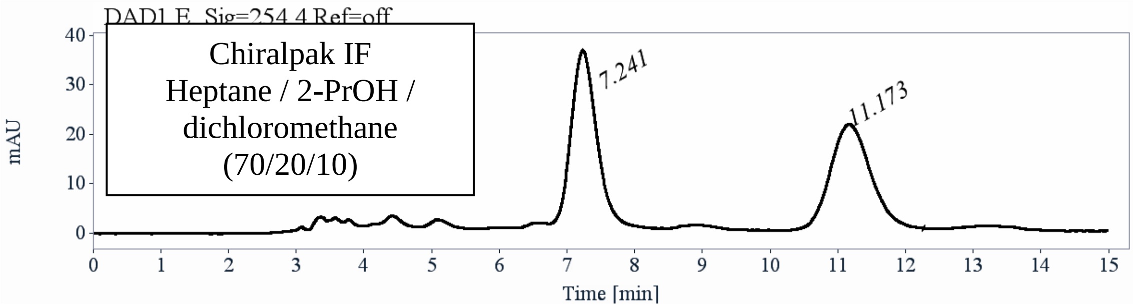

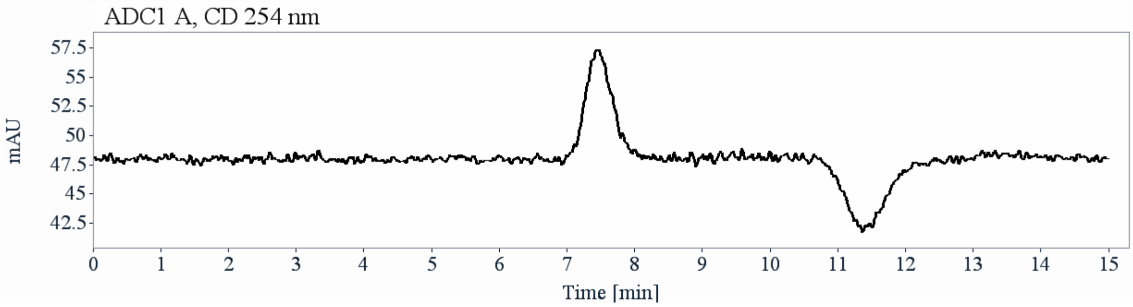

RT [min]	Area	Area%	Capacity Factor	Enantioselectivity	Resolution (USP)
7.24	983	52.49	1.45		
11.17	890	47.51	2.79	1.92	4.44
Sum	1873	100.00			

Chiral HPLC separation for compound 2

Preparative separation:

• Sample preparation: About 24 mg of compound 2 are dissolved in 8 mL of a mixture dichloromethane and ethanol (75/25).

• Chromatographic conditions: Chiralpak IF (250 x 10 mm), hexane / 2-PrOH / dichloromethane (65/20/15) as mobile phase, flow-rate = 5 mL/min, UV detection at 254 nm.

• Injections (stacked): 80 times 100 µL, every 9.4 minutes.

• First fraction: 9.6 mg of the first eluted with ee > 99.5 %

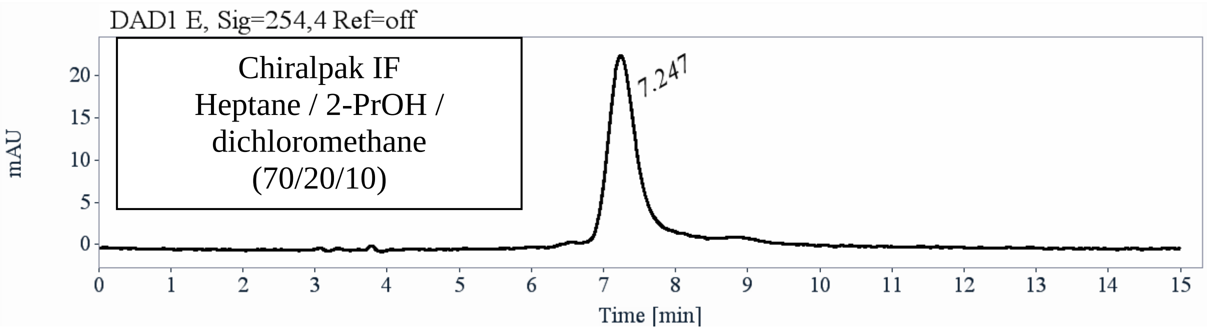

RT [min]	Area	Area%
7.25	626	100.00
Sum	626	100.00

• Second fraction: 9 mg of the second eluted with ee > 99.5 %

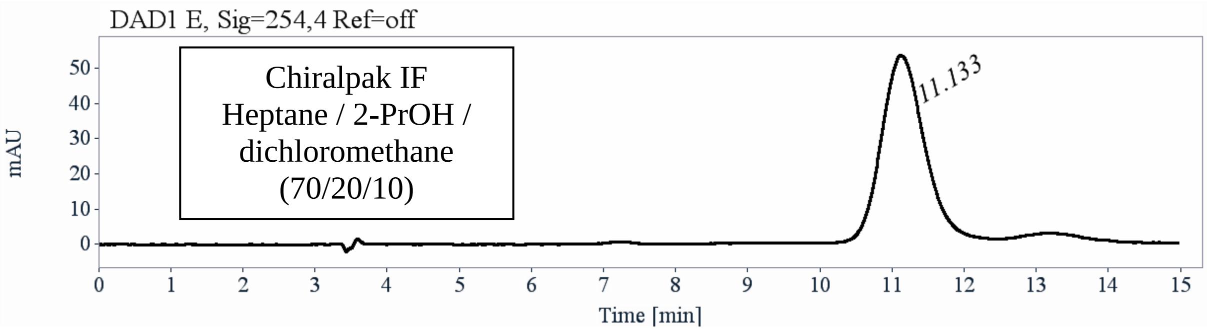

RT [min]	Area	Area%
11.13	2173	100.00
Sum	2173	100.00

See Figure S20 for configurational stability.

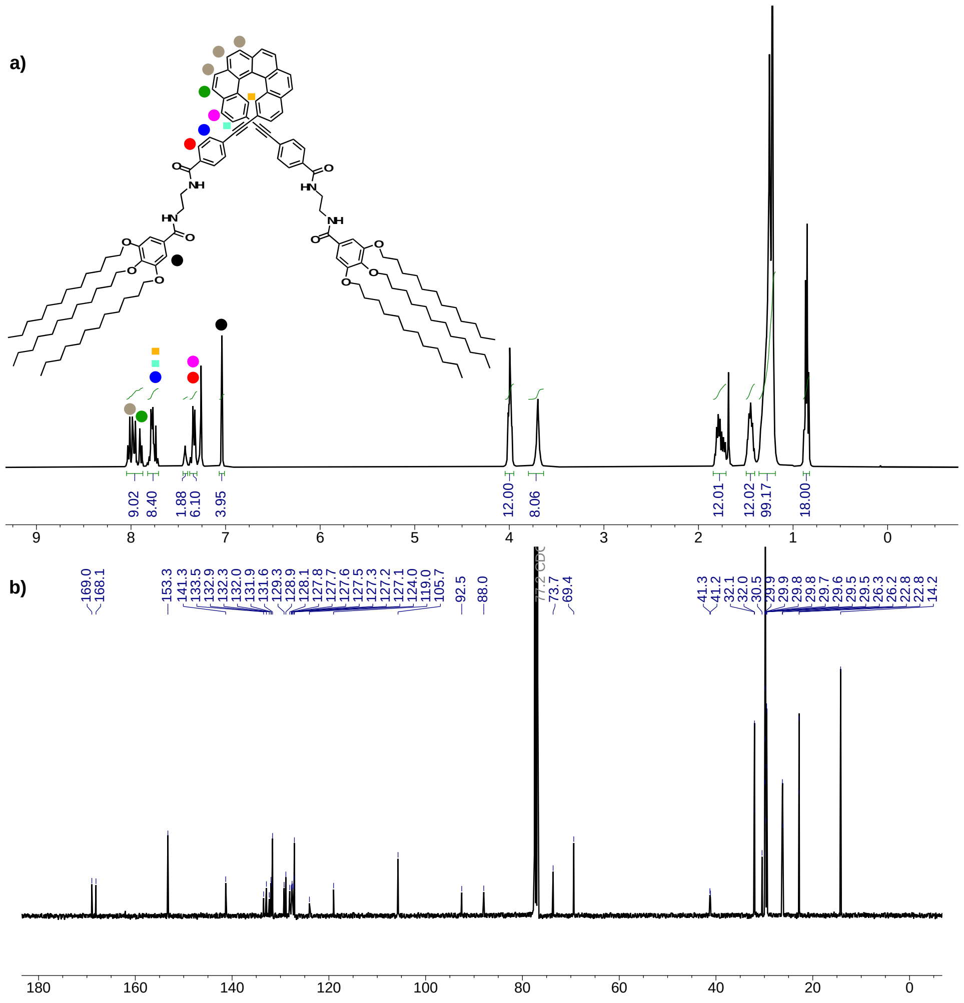

Figure S1. ^{1}H and ^{13}C NMR spectra of **1** ($CDCl_3$, 400 and 100 MHz, respectively)

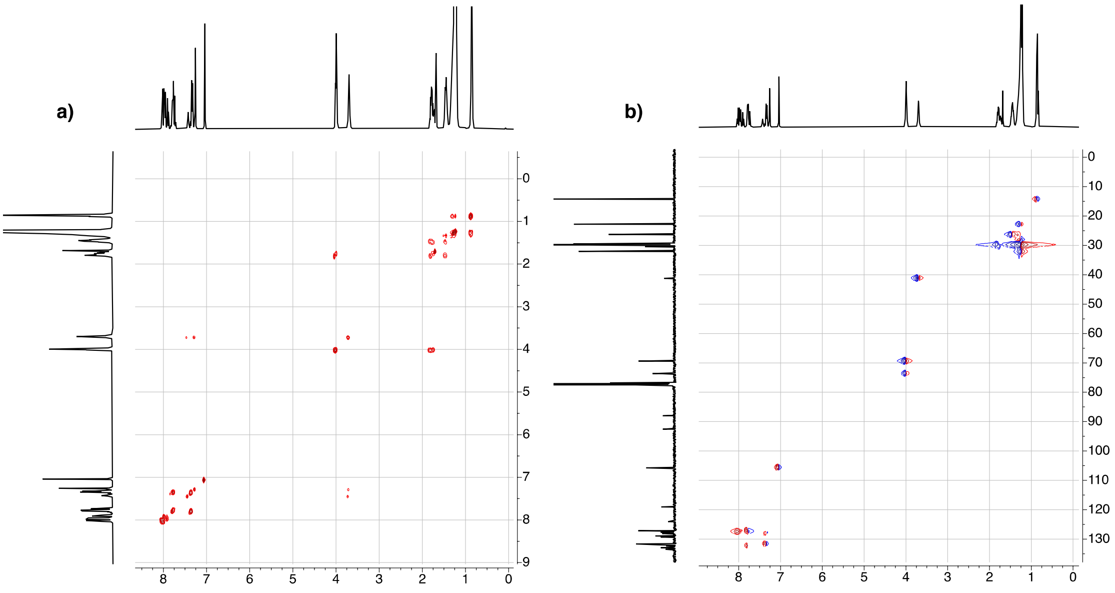

Figure S2. a) ^{1}H-^{1}H COSY and b) HSQC NMR spectra of **1** ($CDCl_3$, 400 and 100 MHz, respectively.)

Concentration Dependent NMR Studies for the assembly of 1

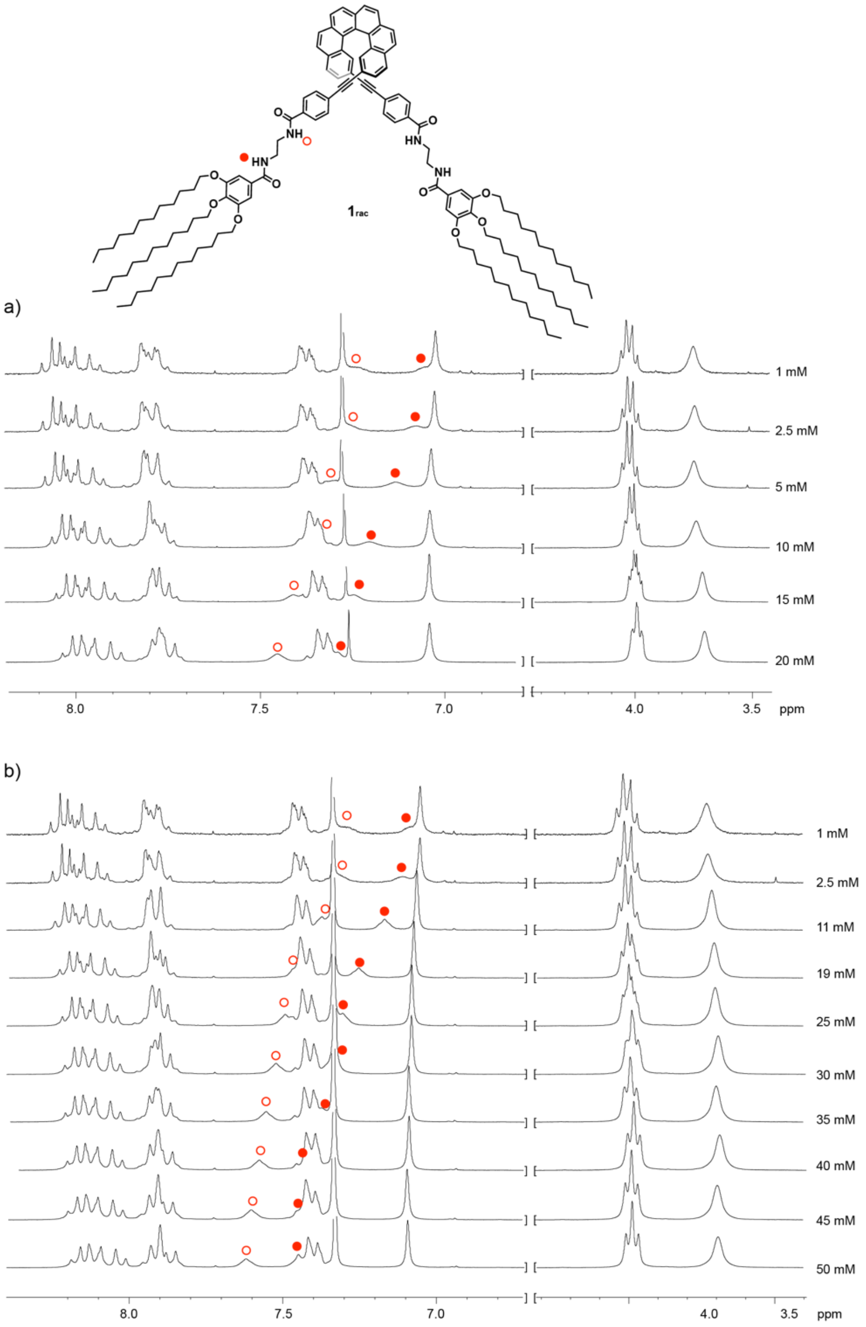

Figure S3. Partial ^{1}H NMR spectra of $\mathbf{1}_{\mathbf{rac}}$ (a) and ***(M)*-1** (b) recorded at different concentrations showing the aromatic and some of the aliphatic protons ($CDCl_3$, 300 MHz, 298 K). The upper part of the Figure displays the chemical structure of [6]helicene $\mathbf{1}_{\mathbf{rac}}$ highlighting the inner (•) and outer (o) amide protons.

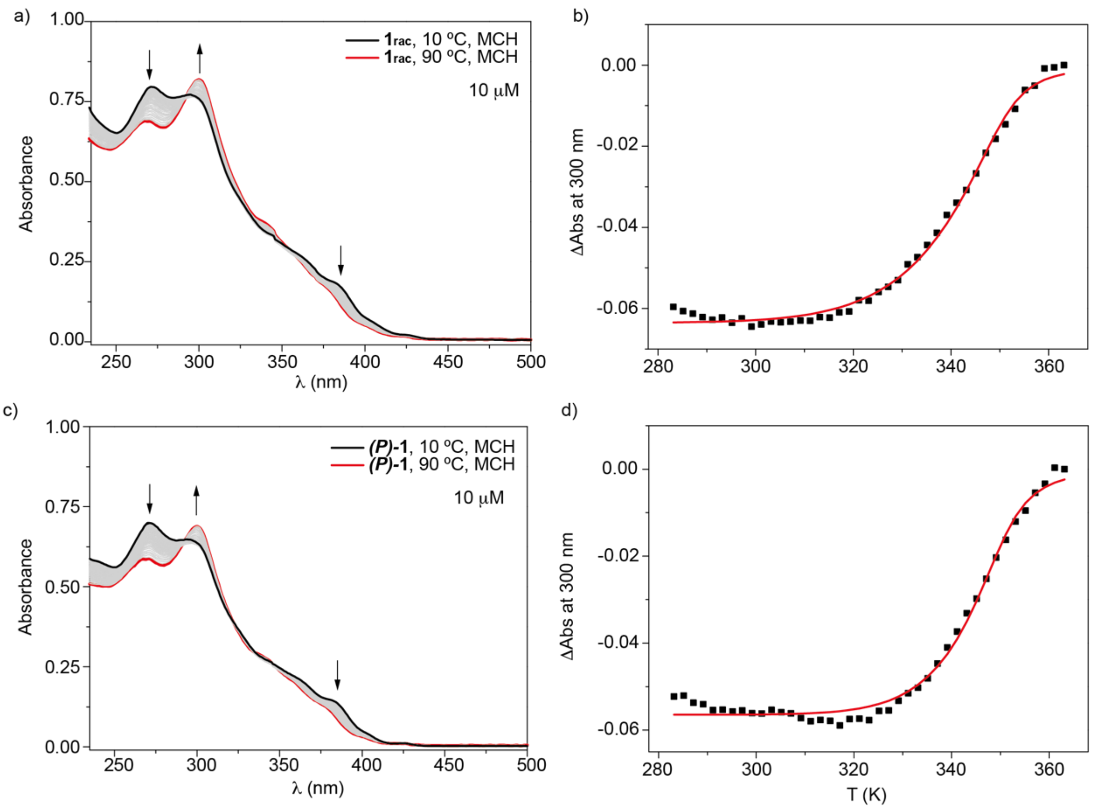

Figure S4. UV-Vis spectra of $\mathbf{1_{rac}}$ (a) and ***(M)*-1** (c) in MCH at c_T = 10 μM. Cooling by plotting the variation of the absorbance at λ = 300 nm for $\mathbf{1_{rac}}$ (b) and ***(M)*-1** (d). The red lines in panels (b) and (c) depict the fitting to the one-component EQ model for supramolecular polymerization. The black arrows indicate the changes observed in the absorption pattern upon increasing the temperature.

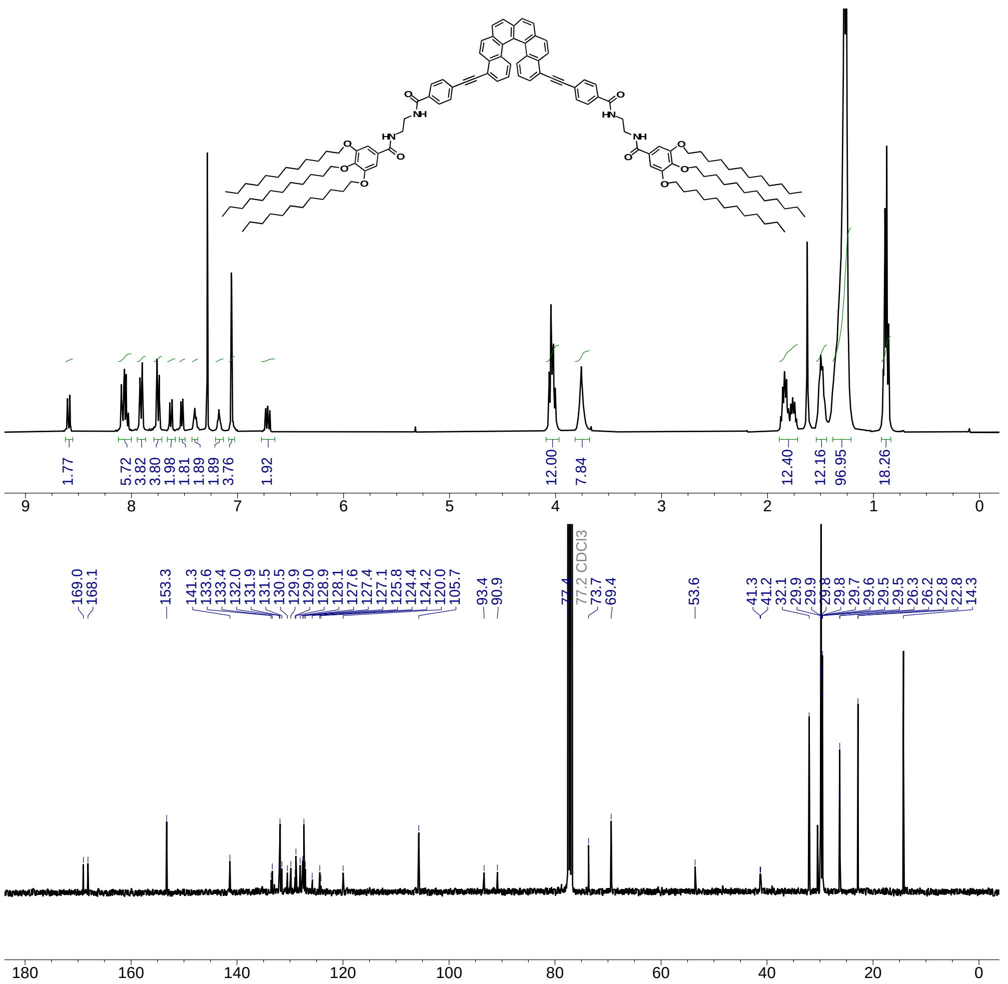

Figure S5. ^{1}H and ^{13}C NMR spectra of **2** ($CDCl_3$, 400 and 100 MHz, respectively).

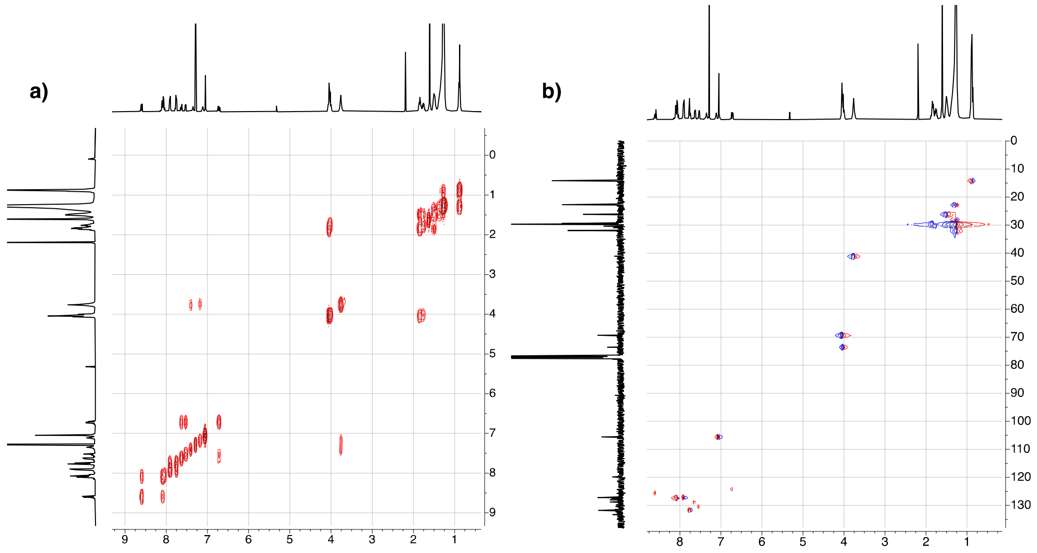

Figure S6. a) ^{1}H-^{1}H Cosy and b) HSQC NMR spectra of **2** ($CDCl_3$, 400 and 100 MHz, respectively.)

NMR Demonstration of the Intramolecular H-bonding: 7-membered pseudocyle formation

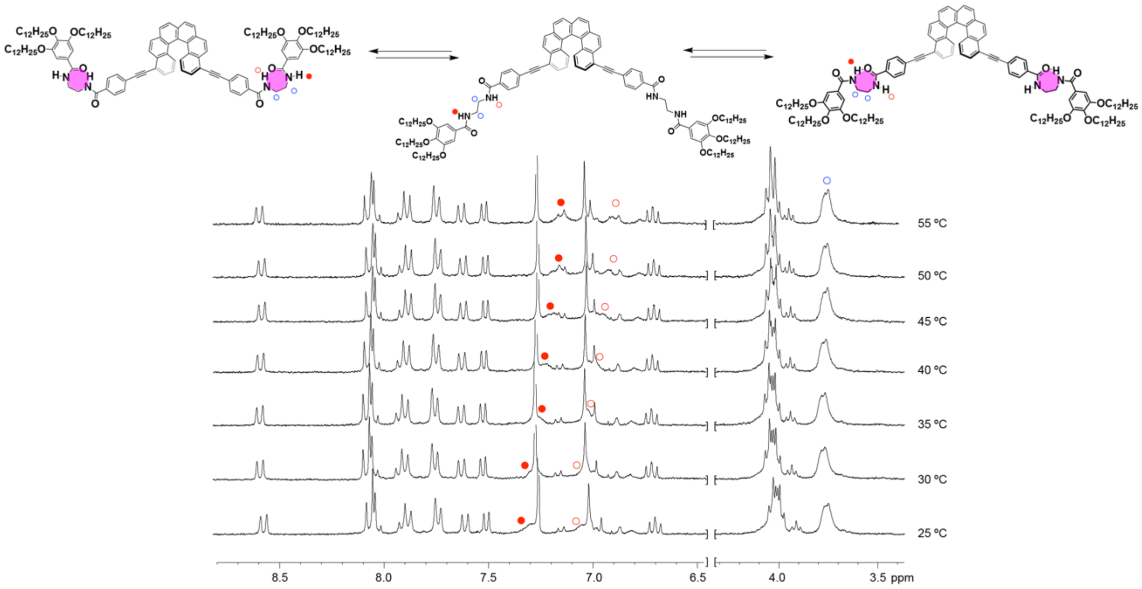

Figure S7. Temperature dependent ^{1}H-NMR spectra of ***(M)*-2** ($CDCl_3$, 400 MHz).

Concentration Dependent NMR Studies for the assembly of 2

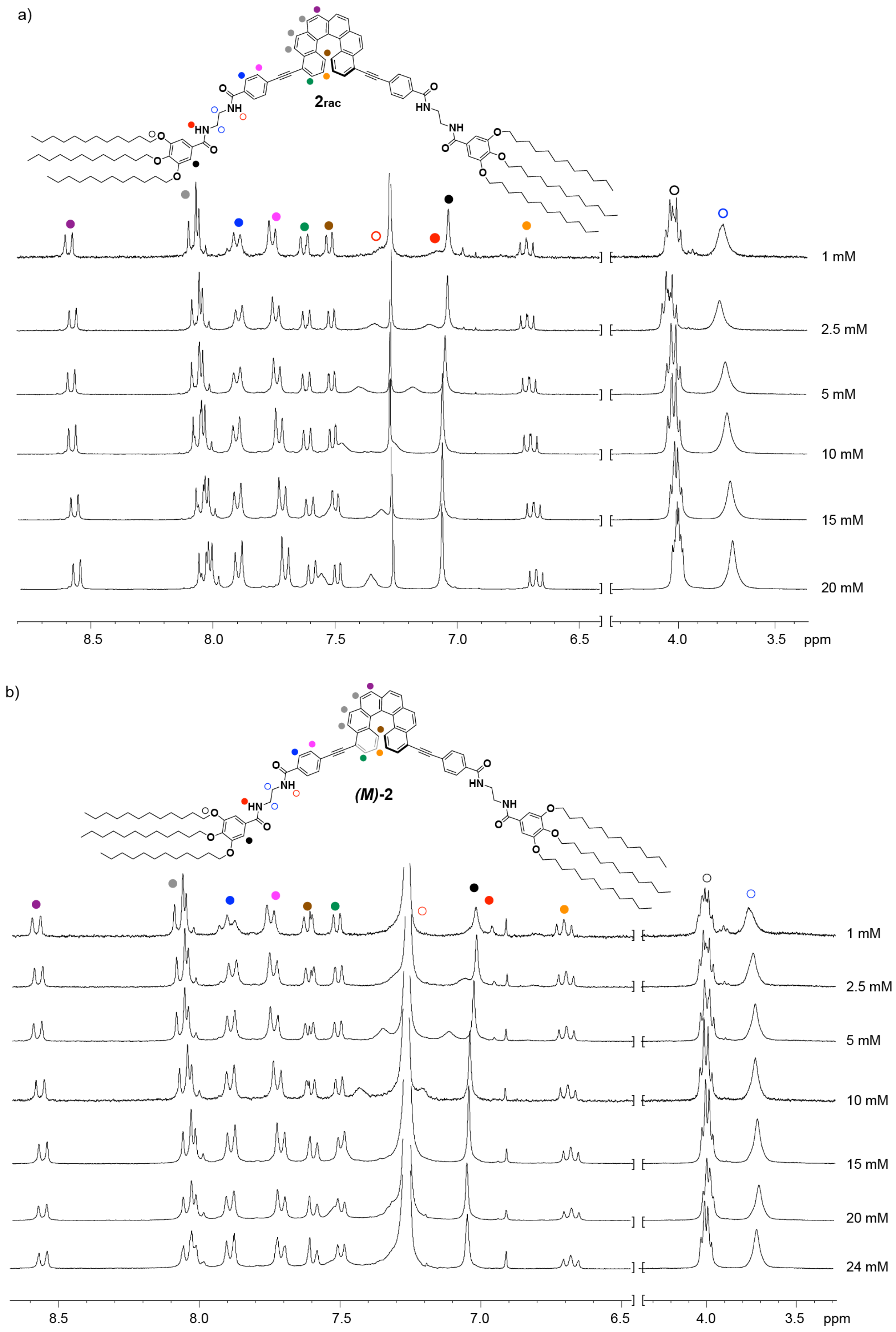

Figure S8. Partial ^{1}H NMR spectra of $\mathbf{2}_{rac}$ and ***(M)*-2** registered at different concentrations showing the aromatic and some of the aliphatic protons ($CDCl_3$, 300 MHz, 298 K).

Additional Spectroscopic studies

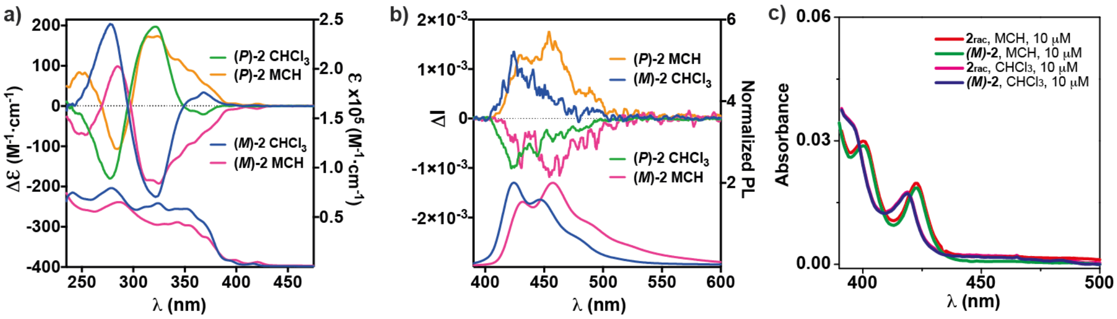

Figure S9. Comparison of (a) ECD/UV-Vis and (b) CPL/PL spectra of **(*P*)-2** and **(*M*)-2** (10 μM, $CHCl_3$ and MCH). (c) Expansion of the UV-Vis response.

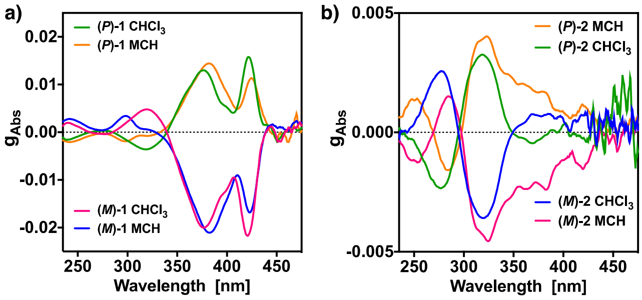

Figure S10. Comparison of the g_{abs} of (a) **1** and (b) **2** derivatives.

Additional AFM Experiments

AFM experiments were carried out to visualize the morphology of the supramolecular assemblies generated by **1** and **2**. Sample was prepared by spin-coating 10 μM solution of the corresponding assembly onto freshly cleavage of HPOG. The results are summarized below.

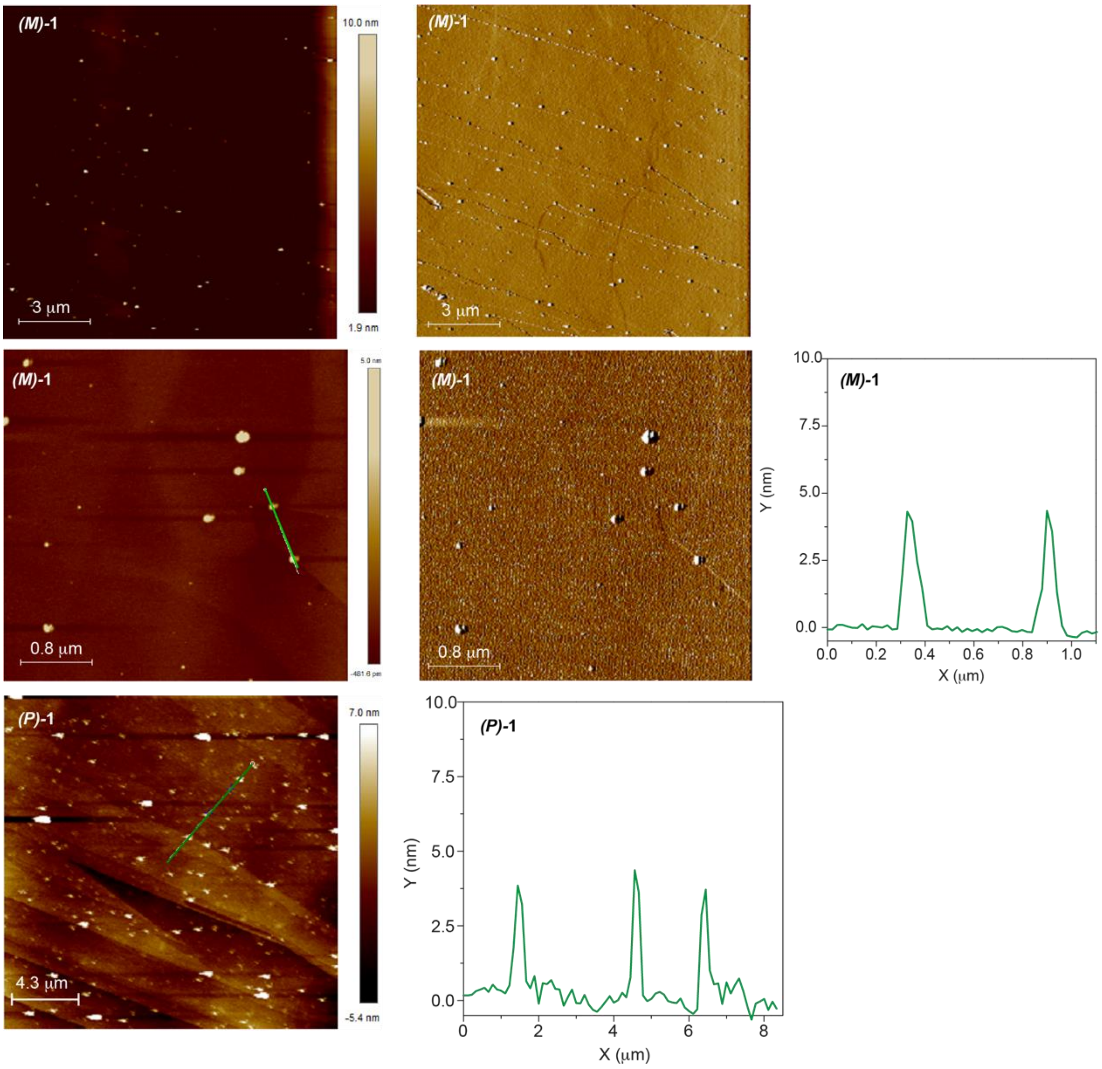

Figure S11. AFM studies for **(*P*)-1** and **(*M*)-1.** Experimental conditions: HOPG as surface, MCH, c_T = 10 μm.

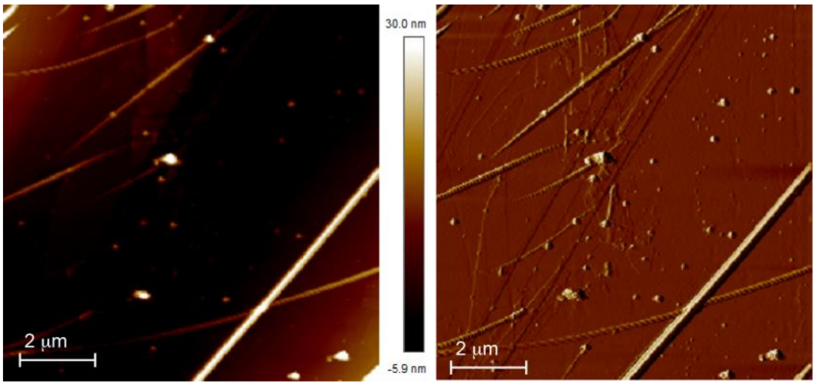

Figure S12. AFM images of the racemic mixture of **(*P*)-2** and **(*M*)-2.** Experimental conditions: HOPG as surface, MCH, c_T = 10 μm.

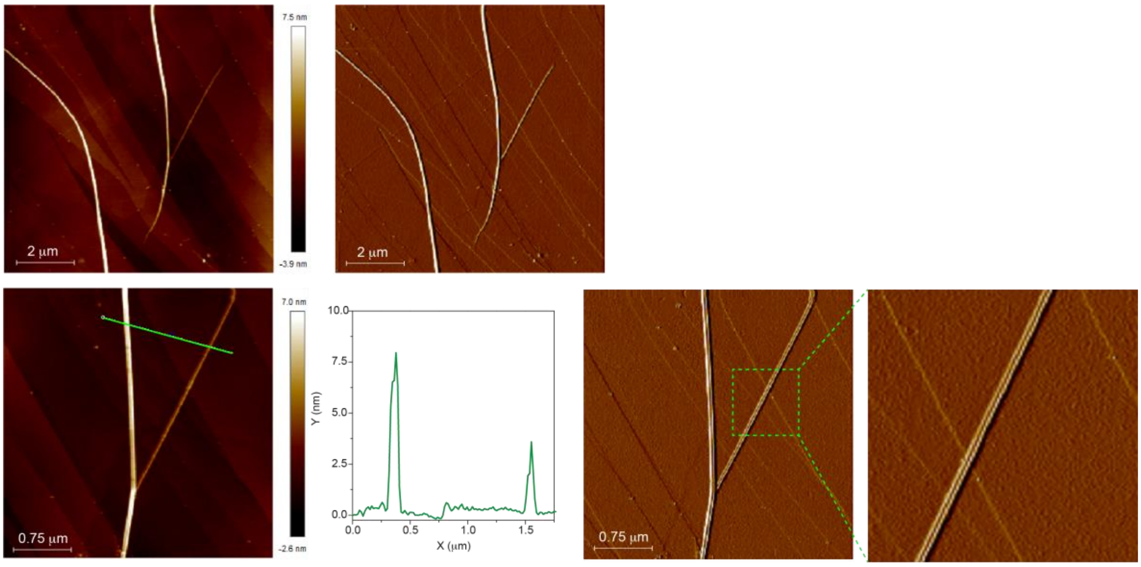

Figure S13. AFM studies for ***(M)*-2**. Experimental conditions: HOPG as surface, MCH, c_T = 10 μm.

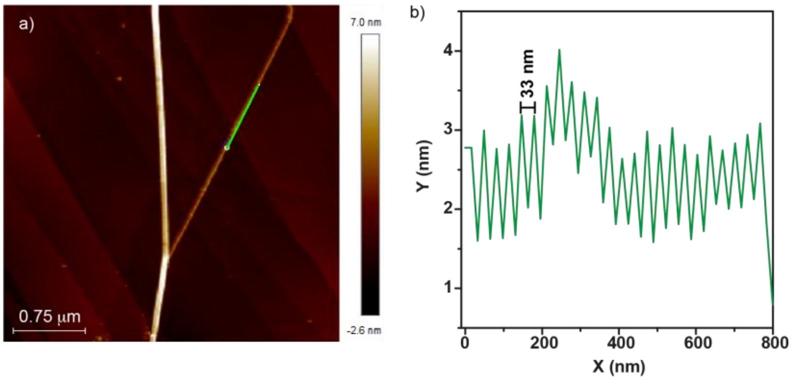

Figure S14. Height AFM image (a) and height profile (b) along the green line in panel (a) of a helical fiber formed by ***(M)*-2** showing the helical pitch. Experimental conditions: HOPG as surface, MCH, c_T = 10 μm.

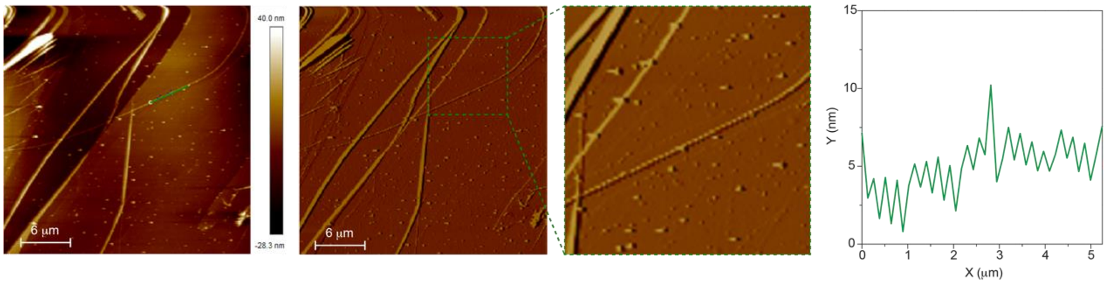

Figure S15. Height and phase AFM images of the helical fibers formed by ***(P)*-2**. The height profile corresponds to the green line in the left panel image. Experimental conditions: HOPG as surface, MCH, c_T = 10 μm.

Atomic force microscopy with magnetic conducting probe (mc-AFM):
Sample preparation for magnetic- conductive atomic force microscopy (mc-AFM):

The substrates for the mc-AFM studies were prepared using the thermal evaporation deposition technique. A 100 nm layer of Ni layer is sputtered, followed by a 10 nm layer of Au layer on a Si wafer, with a 10 nm Ti layer as an adhesion layer. The deposited multilayer surfaces were cleaned by immersing them first in boiling acetone and then in ethanol for 10 minutes. The surfaces were also cleaned with UVozone for 15 minutes, followed by a final 45-minute incubation in warm ethanol. The role of the Ni/Au surfaces is to allow spin polarization of electrons injected from the surface into the chiral molecules induced by a magnetic field. The molecules were deposited by drop-casting the solutions onto the cleaned surface.

Measurement using mc-AFM:

Magnetic field-dependent current-voltage (*I-V*) characteristics of the prepared samples were determined using a multimodal scanning magnetic probe microscopy (SPM) system equipped with a Beetle Ambient AFM and an electromagnet with R9 electronic controller (RHK Technology). Voltage spectroscopy for the *I-V* measurements was performed by applying voltage ramps with a non-magnetic Pt tip (DPE-XSC11, μmasch with spring constant 3-5 Nm^{-1}) in contact mode.

Prior to the current versus voltage (*I-V*) studies, the morphology of the samples was analyzed using AFM topography images. The corresponding images are shown in Figures 6b and S16. The figures clearly show that the **(*P*)-1** and **(*M*)-1** samples form aggregates, while the **(*P*)-2** and **(*M*)-2** samples form helical nano fibers. At least 50 *I-V* curves were scanned in an applied magnetic field of 0.50 T for both magnetic field orientations (field UP and DOWN) and representative plots for **(*P*)-1** and **(*M*)-1** and **(*P*)-2** and **(*M*)-2** samples are shown in Figure S17 and S18, respectively.

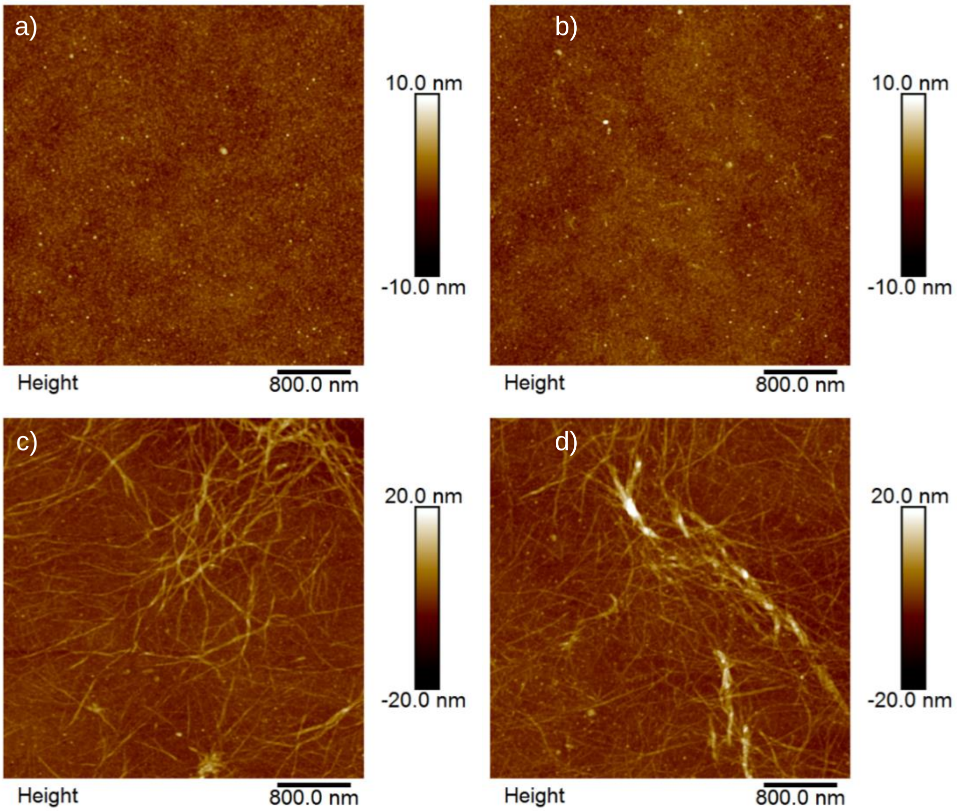

Figure S16: AFM topography images for the **(*M*)-1** (a), **(*P*)-1** (b), **(*M*)-2** (c) and **(*P*)-2** (d) samples on the gold-coated nickel substrate. The figures clearly shows that **(*P*)-1** and **(*M*)-1** samples form globular aggregates, while the **(*P*)-2** and **(*M*)-2** samples form helical nano fibers on the Ni/Au surface.

Spin dependent transport properties for *(M)*-1 and *(P)*-1 enantiomers:

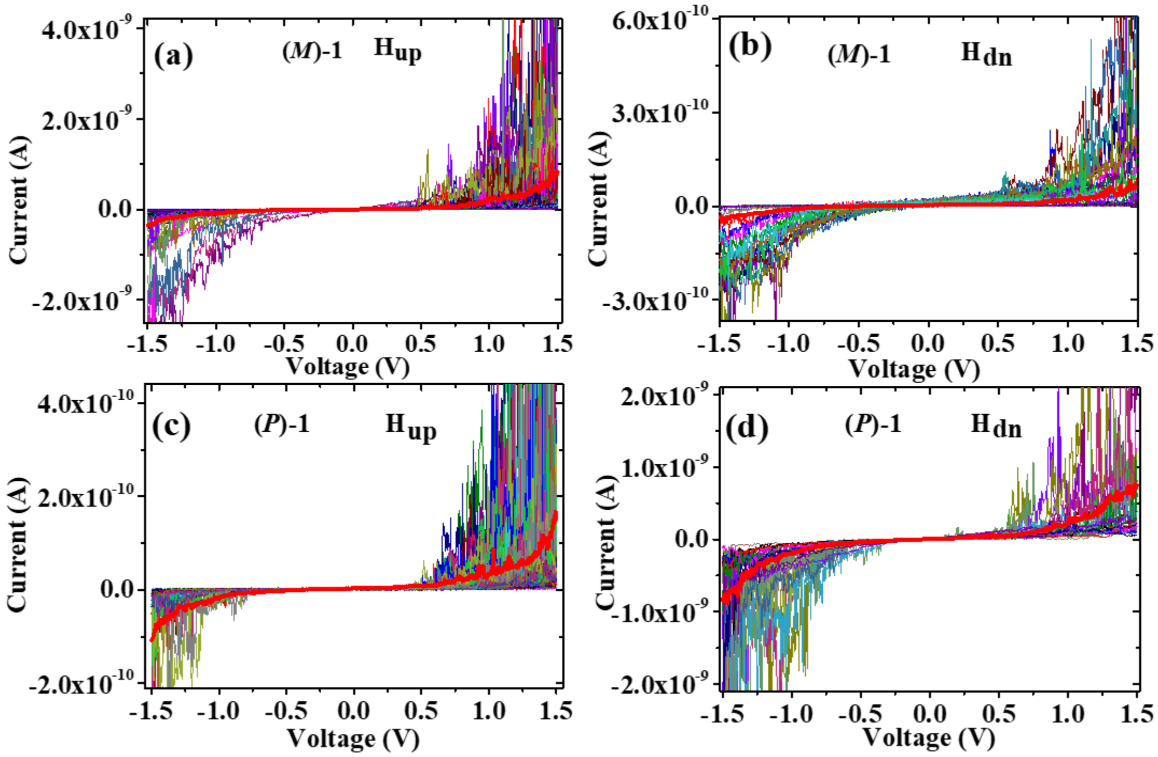

Figure S17: Spin dependent transport properties for ***(M)*-1** and ***(P)*-1** enantiomers with the magnet north pole pointing up (a&c) or down (b&d) respectively. At least 50 *I*-*V* curves were scanned in an applied magnetic field of 0.50 T for both magnetic field orientation (field UP and DOWN). The bold red curve represents the average over more than 50 *I*-*V* curves.

Spin dependent transport properties for *(M)*-2 and *(P)*-2 enantiomers:

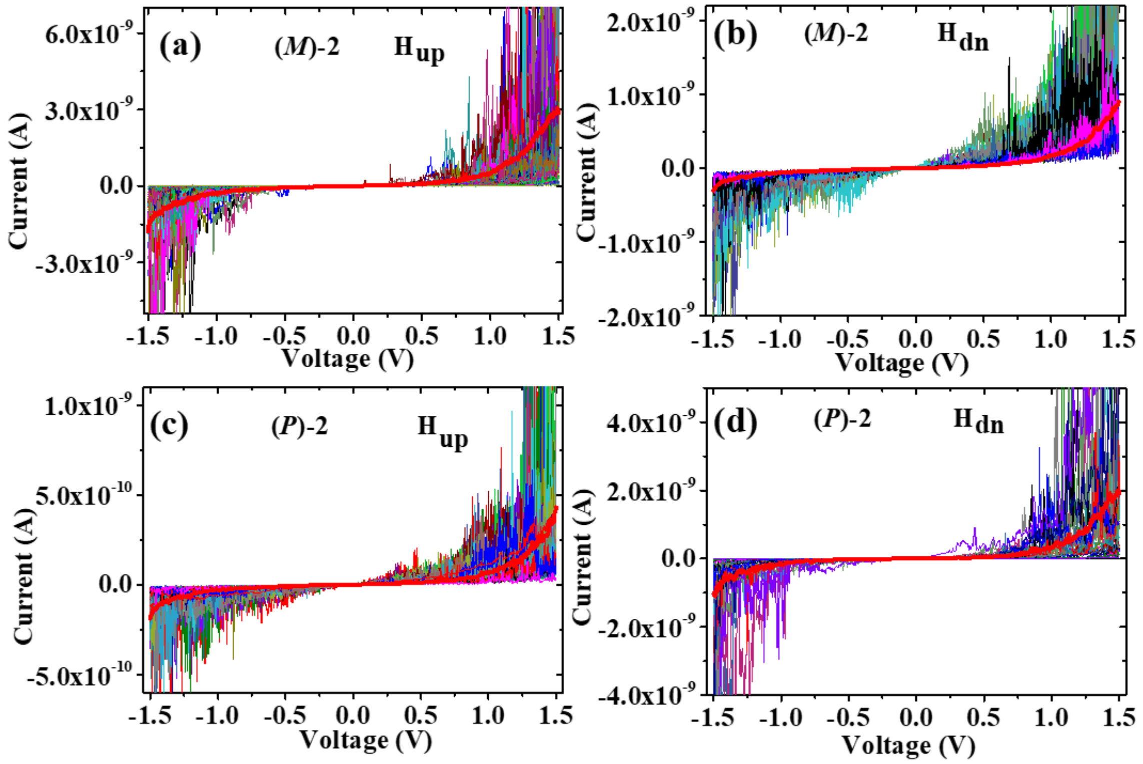

Figure S18: Spin dependent transport for ***(M)*-2** and ***(P)*-2** enantiomers with the magnet north pole pointing up (a&c) or down (b&d) respectively. The bold red curve represents the average over more than 50 *I-V* curves.

Magnetoresistance measurements

A Crossbar configuration was used for the magnetoresistance (MR) device that was produced as described in ref. 11a (Figure 8a). Molecules were spin coated on the top of bottom electrode. On the top of polymer film an insulating buffer layers of 1.5 nm magnesium oxide (MgO) were grown by e-beam evaporation followed by Ni and Au having a thickness of 40nm and 20nm, respectively, using a shadow mask with a line width of ~20μm. The device was subsequently attached to a cryogenic chip carrier and electrically connected by wire bonder (Au wire). The sample was measured by 2T-cryogenics system (Cryogenics Ltd) with different temperature. A magnetic field of up to 1T was applied along the direction of current *i.e.* perpendicular to the sample plane. The resistance of the device was measured using standard four-probe method. DC current of 0.1 mA was applied using a Keithley current source (Model 2400) and the voltage across the junction was measured using a Keithley nanovoltmeter (Model 2182A).

Solid State ECD and UV-Vis studies

The samples were dissolved in MCH at concentrations around 10 μM solution and deposited onto quartz subsrates. The UV-Vis and ECD spectra were measured at different rotating angles and from front and back sides resulting in average spectra.

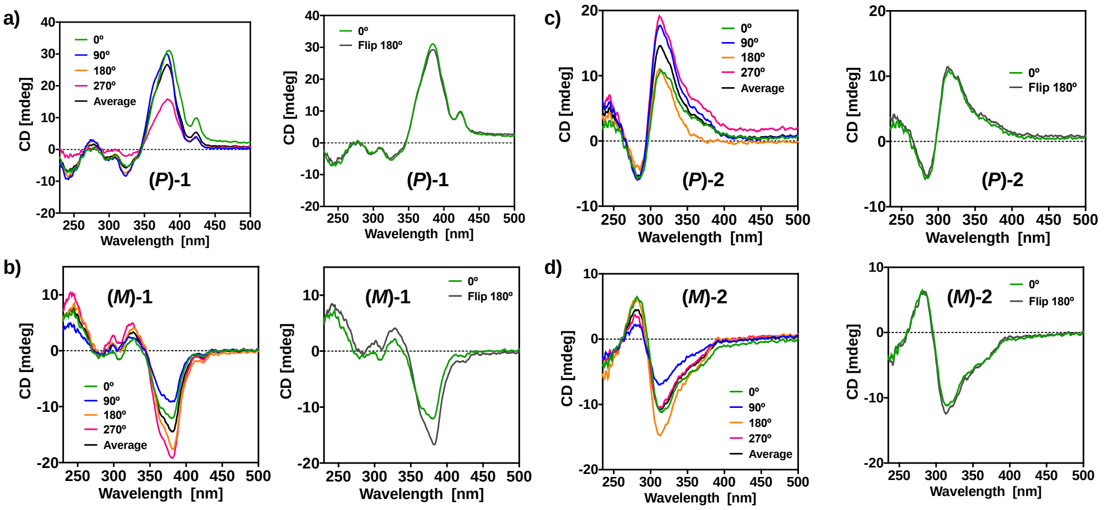

Figure S19. Recorded and average ECD spectra of films deposited on quartz substrates.

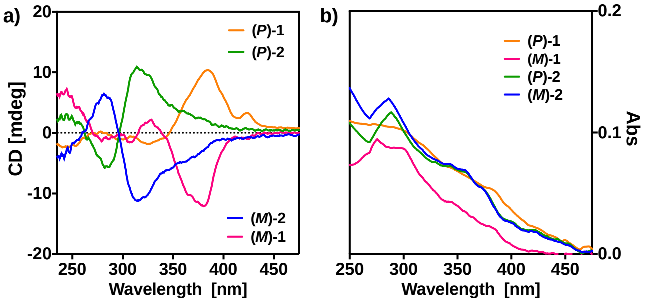

Figure S19b. Average ECD and UV-Vis spectra of films deposited on quartz substrates.

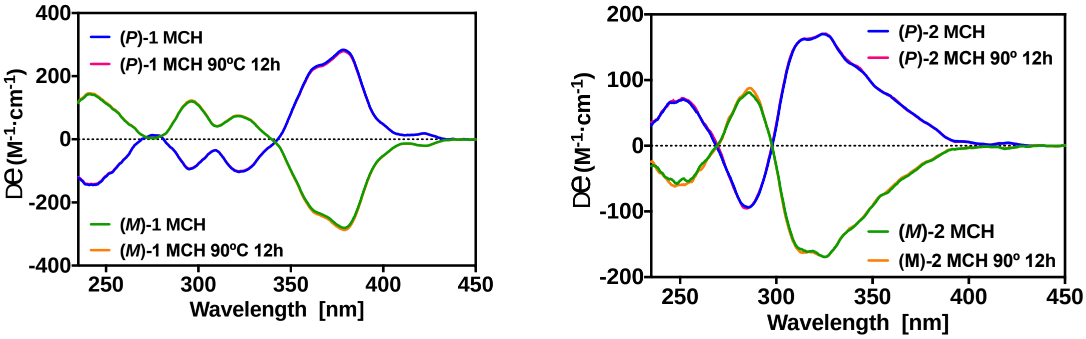

Figure S20. Tests of racemization upon heating in MCH for 12 hours.

References

(S1) Korevaar, P. A.; Schaefer, C.; Greef, T. F. A. De; Meijer, E. W. Controlling Chemical Self-Assembly by Solvent-Dependent Dynamics. *J. Am. Chem. Soc.* **2012**, *134*, 13482−13491.

(S2) Malik, A. U.; Gan, F.; Shen, C.; Yu, N.; Wang, R.; Crassous, J.; Shu, M.; Qiu, H. *J. Am. Chem. Soc.*, **2018**, *140*, 2769-2772.

(S3) Dhbaibi, K.; Favereau, L.; Srebro-Hooper, M.; Quinton, C.; Vanthyune, N.; Arrico, L.; Roisnel, T.; Jamoussi, B.; Poriel, C.; Cabanetos, C.; Autschbach, J.; Crassous, J. *Chem. Sci.*, **2020**, *22*, 567-576.

(S4) Aparicio, F.; García, F.; Sánchez, L. *Chem Eur J.*, **2013**, *19*, 3293-3248.

(S5) Das, S.; Okamura, N.; Yagi, S.; Ajayagosh, A. *J. Am. Chem. Soc.*, **2019**, *141*, 5635-5639.
